# Bacterial intra-species gene loss occurs in a largely clocklike manner mostly within a pool of less conserved and constrained genes

**DOI:** 10.1038/srep35168

**Published:** 2016-10-13

**Authors:** Evgeni Bolotin, Ruth Hershberg

**Affiliations:** 1 Rachel & Menachem Mendelovitch Evolutionary Processes of Mutation & Natural Selection Research Laboratory, Department of Genetics and Developmental Biology, the Ruth and Bruce Rappaport Faculty of Medicine, Technion-Israel Institute of Technology, Haifa 31096, Israel

## Abstract

Gene loss is a major contributor to the evolution of bacterial gene content. Gene loss may occur as a result of shifts in environment leading to changes in the intensity and/or directionality of selection applied for the maintenance of specific genes. Gene loss may also occur in a more neutral manner, when gene functions are lost that were not subject to strong selection to be maintained, irrespective of changes to environment. Here, we used a pangenome-based approach to investigate patterns of gene loss across 15 bacterial species. We demonstrate that gene loss tends to occur mostly within a pool of genes that are less constrained within species, even in those strains from which they are not lost, and less conserved across bacterial species. Our results indicate that shifts in selection, resulting from shifts in environment are not required to explain the majority of gene loss events occurring within a diverse collection of bacterial species. Caution should therefore be taken when attributing differences in gene content to differences in environment.

Gene content varies greatly between bacteria. In comparative genomic studies gene content variation is often attributed to environmentally driven natural selection (e.g. refs 1, 2, 3, 4). A major force, shaping gene content variation is loss of gene functions^5^,^6^,^7^,^8^ (which we will refer to from now on as gene loss). Two competing hypotheses exist with regards to the reasons driving gene loss. First, it is possible that most genes are lost because of shifts in natural selection driven by changes in the environment. Under such a model genes may be lost, following a change in environment, because while they were important in the previous environment, they have now become harmful to fitness in the new environment^9^,^10^,^11^,^12^,^13^,^14^. This will lead to positively selected gene loss. Environmental shifts can also lead to gene loss through relaxations in purifying selection, when a gene is important in other environments, but loses its importance in a particular environment and can therefore be lost from the genomes of bacteria residing within that particular environment^15^,^16^,^17^,^18^,^19^. A second, more neutral, possibility is that most of the genes lost are ones that, to begin with, were less crucial within their species and across species, independent of environment. Under such a model most genes lost are lost irrespectively of any shift in natural selection. Rather, across environments, weaker selection for the maintenance of these genes makes their loss subject to the stochastic whims of genetic drift.

If indeed, gene loss is most frequently driven by genetic drift, rather than by shifts in natural selection, we would expect it to occur in a largely clocklike manner, both within and between species. Previous studies demonstrated that gene loss between bacterial species indeed occurs in a clocklike, time-dependent manner^20^,^21^. However, it is still not clear whether gene loss also occurs in a time-dependent manner within bacterial species.

If the majority of lost genes are lost neutrally, because to begin with maintenance of their functions was less constrained by selection, it would be expected that genes that are lost will tend to be ones that are less constrained in evolution even prior to their loss. We would therefore expect, under a model by which most gene loss is driven by drift, to observe a pattern by which genes that have been lost by certain members of a species, will tend to be less conserved across species and less constrained among those members of the species in which they are still maintained. Some evidence has been found for eukaryotes indicating that genes that are less conserved between species are lost more often between species^22^,^23^. Whether this is also true within species of bacteria remains to be tested.

A useful tool for identifying putative gene loss instances is the pangenome. The pangenome is the collection of all genes found within an investigated bacterial lineage (usually a “species”)^24^,^25^,^26^. To construct a pangenome, orthologous genes are combined into clusters, referred to as pangenes. Once we cluster orthologs into pangenes, it is possible to draw a pangenome plot depicting the distribution of frequencies with which different pangenes are found within the species. The typical pangenome plot is asymmetrically U-shaped, with a much higher frequency of pangenes found either in a few or the majority of the investigated strains and a relatively low frequency of pangenes found in an intermediate number of strains^27^,^28^,^29^. This U-shaped distribution of pangene frequencies suggests that pangenes found in all or a majority of strains of a species are likely vertically inherited within that species^29^. Such universally or nearly universally present pangenes are not likely to have arisen via HGT, as this would require that genes introduced via HGT very often rise to high frequencies among members of a species, but almost never to intermediate frequencies. The U-shape of the pangenome plot also indicates that unique and rare pangenes are likely introduced by horizontal gene transfer (HGT)^29^. After all, explaining the presence of rare pangenes via gene loss would require a very high number of gene loss events and, again, require that genes often be lost many times, but rarely be lost an intermediate number of times. Supporting our claim that unique and rare pangenes are the ones that are horizontally transferred into a species, we have previously shown that clonal pathogen species that undergo little or no HGT have almost no unique or rare pangenes^29^.

If indeed genes found in the majority or all strains of a species tend to be ones that are vertically inherited within that species, it follows that genes found in most but not all members of the species were lost from those members of the species from which they are absent. It is therefore possible to identify genes that have likely experienced gene loss by considering those genes that are found in most but not all strains of a pangenome. We have previously demonstrated the usefulness of this approach for studying the dynamics of gene loss within a diverse array of bacterial species^29^. At the same time, the pangenome approach can only be used to identify gene loss events. It is less useful for ascertaining how many times a certain gene was lost within a given species. After all, a gene absent from two strains within a species may have been lost once in the common ancestor of these two strains and may have also been lost independently twice. To examine whether a gene was lost once or multiple times it is useful to generate phylogenetic trees and place putative gene loss events on their branches.

Here, we analyze the pangenomes and phylogenetic trees of a large collection of bacterial pathogenic species and compare patterns of conservation and constraint of genes that are lost within each species to those that show no instances of being lost. We demonstrate that across bacterial species genes that are lost tend to be less constrained during the evolution of the species in question and less conserved across other species. Furthermore, genes that are lost multiple times are even less constrained and conserved than genes lost only once. Finally, fitting with previous results showing that gene loss between bacterial species is clocklike^20^,^21^, we find that gene loss is also clocklike within bacterial species. Combined, our results demonstrate that most genes lost by strains of a certain species are lost not because they are no longer required or because there is an adaptive advantage to their loss. Rather most gene loss within bacterial species occurs within a pool of genes that are less conserved and constrained even in those members of the species from which they were never lost and also across other species. This indicates that most lost genes are lost because there is less selection to maintain them making their fate subject more strongly to stochastic (genetic drift) rather than deterministic (natural selection) forces.

## Results

### Identification and classification of gene loss events

We focused on 15 bacterial species for which at least 10 genomes were sequenced (Table S1). Based on the NCBI gene annotations provided with each genome we constructed for each species its pangenome (Materials and Methods). As already discussed in the introduction, pangenes found in most or all genomes of a given species likely represent the vertically inherited gene content of that species, while rare pangenes were likely horizontally introduced. Pangenes found in most but not all members of a species were likely lost from the members of the species in which they are not present. We therefore classify as pangenes that have experienced some gene loss those pangenes that are found in most (75%) but not all members of a species (termed ‘near core’ pangenes). In contrast, those pangenes found in all members of a species (termed ‘core’ pangenes) are classified as unlost (at least in those members of the species that have been sequenced)).

While the pagenome is useful for classifying pangenes into those that have been lost and those that have not, it cannot be used to ascertain how many times a pangene was independently lost within a species (see Introduction). To ascertain how many times each ‘near core’ pangene was lost within in a species we therefore constructed a phylogenetic tree of each of the considered species (Materials and Methods, Figure S1). This allowed us to place gene loss events on the branches of these trees, which in turn allowed us to predict the number of times each ‘near core’ pangene experienced a loss event within its species. This enabled us to classify near core pangenes into ‘single loss’ (if they were lost only once along the phylogenetic species tree) and ‘multiple loss’ (if they were lost on multiple branches of the tree) (Table 1).

### Lost genes tend to be less conserved, less constrained and less biased in their codon usage than genes that have not been lost

**Figure 1:**
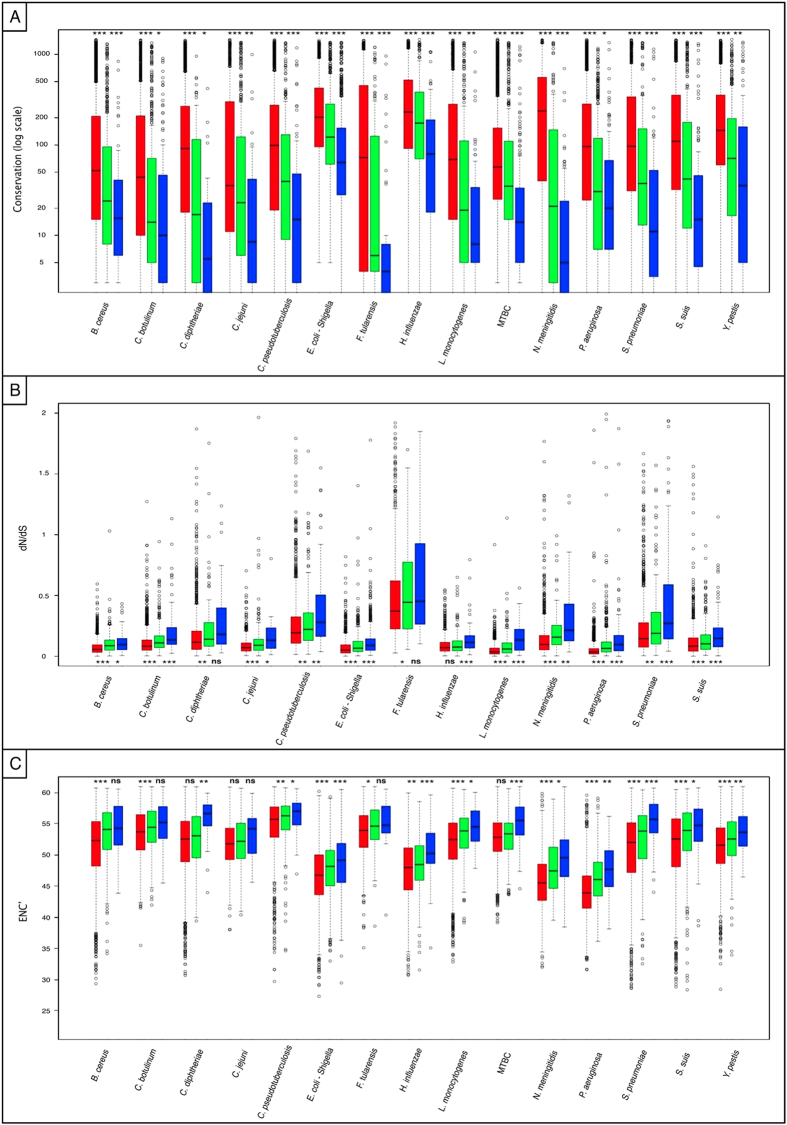
Gene loss occurs mostly within a pool of less conserved and constrained pangenes. Depicted are boxplots representing levels of conservation (**A**), constraint (**B**), or codon bias (**C**) applied on core pangenes (red), single-loss pangenes (green) and multiple loss pangenes (blue). Whisker length for each boxplot represent 1.5 IQR. Statistical significance of differences between the gene-loss groups according to a non-paired, one-sided Mann-Whitney-Wilcoxon test is denoted by: (***) for *P* ≤ 0.001, (**) for *P* ≤ 0.01, (*) for *P* ≤ 0.05, and (ns) for *P* > 0.05. (A) Conservation-the number of additional species in which pangenes are found. (the Y-axis is presented in logarithmic scale). Genes lost multiple times tend to be conserved in less species than genes lost once, which are in turn less conserved than core genes that were never lost. (**B**) constraint within the species the pangenes belong to, as measured using the dN/dS metric. Lower dN/dS values indicate higher constraint. Multiple loss pangenes tend to be less constrained within their species than single loss genes that are in turn less constrained than genes that were never lost. (**C**) Codon Bias, as measured using the ENC’ metric. Lower ENC’ values indicate higher codon usage bias. Core genes that were never lost tend to be more biased in their codon usage than genes that were lost once. Genes that were lost once tend to be more codon biased than genes lost multiple times.

Next, we compared core pangenes, single loss pangenes and multiple loss pangenes with regards to their conservation. To do so, we conducted FASTA^30^ comparisons of each pangene against all fully sequenced bacterial genomes contained within the NCBI database and calculated for each pangene the number of species in which it was present (Materials and Methods). We found that across all species, core pangenes are significantly more conserved than single loss pangenes (*P* ≤ 0.0008, according to a non-paired, one-sided Mann-Whitney test for all comparisons, Fig. 1A and Table S2). Additionally, ‘single loss’ pangenes were in turn significantly more conserved than ‘multiple loss’ pangenes (*P* ≤ 0.026, for all comparisons, Fig. 1A and Table S2).

Next we examined whether genes that were lost within a species, differed from those that were not lost in the constraint applied on them within each species of question. To do so we used the PAML package^31^,^32^ to calculate for each core and near core pangene its ratio of rates of non-synonymous to synonymous substitutions (dN/dS) within its species. This was done based on the sequences of each pangene in the strains in which it was not lost. A lower dN/dS indicates stronger constraint on the pangene in question. Reliable dN/dS analyses require sufficient sequence variation within the examined genes. Such sequence variation is not found in two of the species in our dataset that we previously showed to be extremely non-diverged in their gene sequences (*Y. pestis* and the MTBC)^29^. We therefore removed theses species from consideration, leaving us with 13 species that were analyzed. We found that in 12 of 13 species genes that were lost once were significantly less constrained than genes that were never lost and in 11 of 13 species genes that were lost multiple times were significantly less constrained than genes lost once (*P* < 0.05, Fig. 1B and Table S3).

Genes that are more constrained tend to also be more biased in their codon usage^33^,^34^,^35^,^36^. We therefore compared codon bias (using the ENC’ measure (see Materials and Methods)) between the three pangene groups. We found that for 12 of 15 species core genes were significantly more biased in their codon usage than single loss genes, which in turn were significantly more biased than multiple loss genes in 11 of 15 species (*P* < 0.05, Fig. 1C and Table S4).

Combined, these results demonstrate that, within bacterial species, gene loss tends to occur mostly within a pool of genes that are less conserved across species, less codon-biased, and less constrained within their own species, prior to their loss event.

### Gene loss tends to occur in a time-dependent manner along branches of a species’ phylogenetic tree

The following analyses require substantial per-gene sequence variation. For this analysis we therefore again removed from consideration the MTBC and *Y. pestis* species that display very little gene-sequence variation^29^. Based on our constructed phylogenetic trees of each species, we could predict the number of gene loss events that occurred along each branch of each of the 13 phylogenetic trees. We then used PAML^31^,^32^ to calculate dS (the ratio of synonymous substitutions per synonymous site) for each branch of each tree. Since synonymous substitutions are expected to be less affected by natural selection, dS provides the best available estimate of the length of time that has passed along each branch. We found a significant correlation between dS and number of genes lost, for 8 of the 13 analyzed species (*P* < 0.05 according to the Spearman Rank test, Table S5). These correlations are impressive considering how noisy a per-branch estimation of dS is likely to be. It therefore seems that, at least in the majority of investigated species, the number of genes lost along each branch of the phylogenetic tree shows a correlation with the amount of time that branch represents (as estimated through dS). This indicates that within the majority of species rates of gene loss tend to be clocklike. Since shifts in natural selection are not expected to occur in such a clocklike manner, this suggests that the majority of genes lost are not lost due to such shifts. Rather, such clocklike behavior of gene loss suggests that much of gene loss is neutral to the extent that it is largely governed by the stochastic whims of genetic drift.

## Discussion

Together, our findings suggest that, within bacterial species, gene loss tends to occur in a fairly clocklike manner, and mostly within a pool of genes that were less conserved and constrained even prior to the loss event. This implies that most genes are lost in a time dependent manner, because even prior to their loss they were subject to weaker selection. This in turn indicates that changes in environment, resulting in changes to selection for the maintenance of genes are not required to explain most gene loss events.

It is important to note that we are not implying that environmental shifts do not contribute to gene loss. There are well-documented cases of adaptive gene loss stemming from environmental pressure. For example, the *ompT* gene that is common in *E. coli* was lost in *Shigella* spp. due to its adverse effect on *Shigella* virulence^14^,^37^,^38^. In our analyses we also encountered highly conserved and constrained genes that, nevertheless, were lost in certain strains. The loss of such genes may represent cases of environmental shift-driven gene loss. However, our results do demonstrate that such cases of gene loss are the minority rather than the majority. Most genes that are lost within species are likely lost, because both within that species and across species their maintenance was subject to less strong selection. Therefore, strong caution should be exercised when ascribing specific gene loss events to changes in the environment. In particular one must take care when deducing that the absence of a gene in certain members of a species and its presence in others is due to an environmental shift that led to a shift in the importance of the gene in question.

Even if environmental shifts do not majorly contribute to gene loss by often rendering certain genes that were previously subject to strong constraint, deleterious or no longer needed, they can still have a strong influence on rates of gene loss. Environmental changes, such as movement towards a more restricted lifestyle, can lead to reductions in effective population sizes (N_e_)^5^,^39^,^40^,^41^. When N_e_ becomes smaller, natural selection becomes less efficient relative genetic drift^5^,^39^,^40^,^41^. This means that when N_e_ is small, gene loss events will have to have a stronger effect on fitness in order to be acted upon by natural selection. Reductions in N_e_ can therefore lead to gene loss. For example, we previously demonstrated increases in gene loss associated with host-restricted pathogenic lifestyles, and especially clonal pathogenic lifestyles^29^,^42^. Such increases in gene loss are not due to changes to the directionality or strength of selection applied on specific genes. Rather they are likely due to changes in the intensity of selection applied on the entire genome, due to reductions in N_e_.

Of relevance, our results apply also to clonal pathogens (see results for *C. pseudotuberculosis* and *F. tularensis*). We have previously demonstrated that such clonal pathogens experience massive gene loss, relative their low levels of gene sequence diversity^29^. It is likely that this massive gene loss occurs due to reduced N_e_ of these clonal pathogens, stemming from near lack of recombination and their pathogenic lifestyle. Our current results show that even within such pathogens that experience relatively high proportions of gene loss, gene loss appears to be time-dependent and that those genes that are lost tend to be less constrained and conserved to begin with.

It appears possible that we do not observe a strong signal of environmentally driven loss of specific genes, because the strains we are considering within each species are simply not diverged enough in their ecological niches. Indeed, for many of the species considered, different strains do seem to reside in quite similar niches (e.g. are all human pathogens infecting the same organ). However, we did investigate a number of species whose strains live in rather variable environments. For example, the sequenced *Escherichia coli* strains we analyzed were isolated from a number of different niches and include commensals, pathogens with different niches (e.g. urinal tract infection vs. enterohemorrhagic), as well as lab strains (Table S1). The *Listeria monocytogenes* species is known to include a number of distinct lineages living in different, though sometimes overlapping ecological niches^43^. *Corynebacterium pseudotuberculosis* strains form two lineages (biovars) specialized for survival in distinct animal hosts^44^. The *Mycobacterium tuberculosis* complex (MTBC) strains analyzed also were shown to infect different hosts with varying success^45^, and include both human and animal isolates (Table S1). It therefore does not seem that lack of environmental differentiation explains our finding that genes that are lost within species tend to be lost in a clocklike manner, mostly from a less constrained and conserved gene pool.

If a gene is lost independently from many strains of given species or subspecies, it may give a reason to suspect that these events represent adaptive convergent gene loss that removes unnecessary or even outright harmful genes from the genetic repertoire of these strains. Indeed, the *cadA gene* that was shown to be subject to adaptive gene loss in *Shigella*, was lost from strains of *Shigella* in a pattern indicative of multiple loss events^13^. However, we show that genes lost multiple times within a species tend to be even less conserved and constrained than genes lost only once. This indicates that most ‘multiple-loss’ pangenes are lost multiple times simply because they are even less conserved and constrained than the ‘single-loss’ pangenes, rather than because of convergent adaptive gene loss. These results demonstrate that caution needs to be taken when using convergent gene loss as a signal of positive selection.

It is important to note that we are not claiming that certain functions do not tend to more often be lost than others. Indeed, a functional classification of “lost” vs. “unlost” pangenes, using the COG database (Materials and Methods) demonstrated that certain COG functions tend to be significantly over-represented among core pangenes that were never lost, compared to near core pangenes that were lost in some members of a species (Table S6). The COGs that are over-represented among pangenes that were never lost include housekeeping type functions such as cell-cycle control, nucleotide metabolism, translation and replication (*P* < 0.05, according to the two-tailed Mann-Whitney test, see Materials and Methods and Table S6). In the other direction, the ‘function unknown’ and ‘defense mechanisms’ COGs were found to be over-represented among lost pangenes, relatively to pangenes that were never lost (*P* < 0.05, according to the two-tailed Mann-Whitney test, see Materials and Methods and Table S6). These trends likely demonstrate that genes belonging to housekeeping categories are more constrained by evolution across species and are therefore less readily lost.

In conclusion, we show that bacterial intra-species gene loss occurs in a largely clocklike manner within a pool of genes that are less conserved and constrained, even prior to their loss and across species. While environmental shifts leading to changes in selection applied on specific gene functions may play a certain role in determining which genes are lost, shifts in environment are not required to explain most instances of gene loss. Caution should therefore be exercised when trying to ascribe differences in gene content, stemming from gene loss, to changes in environment.

## Materials and Methods

### Datasets

Data used in this study were downloaded from the NCBI (National Center for Biotechnology Information) database^46^. Bacterial species were selected for analyses that contained at least 10 fully sequenced members each. Strains, whose genomes were not fully sequenced, strains that were artificially manipulated, and strains that represent multiple clones taken from certain site rather than different strains were removed from further analysis. From the 13 strains of *Clostridium botulinum* available on the NCBI database, only 10 strains belonging to lineage 1 were analyzed. In addition, *Escherichia coli* and *Shigella spp.* datasets were combined into one since studies indicate that *Shigella* strains are members of the *E. coli* species^27^,^47^,^48^. The list of all species and strains analyzed is provided in Table S1. A list of strains removed from consideration is provided in Table S7.

### Pangenome construction

In order to identify cases of gene loss within the investigated organisms, the pangenomes of the investigated bacterial species were constructed, as described in ref. 29. Briefly, to generate a pangenome we selected the genome of one of the strains of the species for which the pangenome was to be constructed at random to serve as the initial library. All remaining strains were then compared iteratively to the library using FASTA^30^. During each pairwise comparison, identified orthologs were combined into pangene groups. The query sequences that didn’t find a match in the library were added to the library, so the comparison of the following genome was carried out against an expanded library. After all the genomes were compared the resulting pangenome was compared to itself to minimize computational bias stemming from random choice of the initial library. The generated pangenome was also corrected for additional possible artifacts as in ref. 29.

Next, each pangene was corrected in regard to the length of its members. We assumed that within relatively closely related strains genes sharing considerable sequence similarity and performing similar function are likely to have somewhat similar length as well. Therefore, if within a given pangene, one gene is considerably shorter than the rest, it is not likely to perform the same function and may not even be functional at all. To determine if any pangene within the pangenome includes short members, the length of all member genes within the pangene was compared to the length of the longest member in the given group. Genes whose length was less than two-thirds of the longest gene were removed from the pangene and the pangene was considered as absent in their respective genomes.

In order to build each pangenome we had to select a threshold for assigning the presence or absence of a pangene within each strain of a species. We sought to select this threshold based on analyzed levels of gene sequence similarity within each of the investigated species. Towards this end, pairwise comparisons of all strains within each species were performed using FASTA^30^. For each pair of strains orthologous gene pairs were identified then as bi-directional best hits in which the length of the shortest gene in the pair was at least two-thirds of the longest gene and the sequences were aligned over at least 70% of the shortest gene length. Next, the distribution of gene sequence identity % among all orthologous gene pairs was calculated. Within all investigated species at least 95% of orthologs were at least 80% identical in their sequences (Table S8). We therefore selected a threshold of 80% sequence identity for clustering orthologous sequences within the pangenomes. Since in each species less than 5% of orthologs have a percent identity lower than the selected threshold, our chances of assigning a pangenome as absent from a certain genome, while it is in fact present but diversified can be said to be lower than 5%.

### Phylogenetic tree construction

Phylogenetic trees were constructed using the neighbor-joining algorithm implemented by the PHYLIP package’s Neighbor program^49^. In order to create rooted trees an outgroup strain outside of each species was selected for each of the 15 examined species. The metric used to estimate the distance between each genome pair within a species (including the outgroup) was average nucleotide dissimilarity (AND) defined as 100 – ANI, where ANI stands for average nucleotide identity. In order to minimize possible effects of recombination, we used only genes belonging to the ‘core’ pangenome that showed no trace of recombination^29^ for calculating ANI and AND.

The ‘core’ non-recombining gene sequences of strains belonging to the same bacterial species were compared in the pairwise manner using FASTA^30^. Orthologous gene pairs were identified as reciprocal best hits. Following the thresholds set by POGO-DB^50^, only putative orthologous gene pairs sharing 30% identity over at least 70% of the gene length were used for ANI calculation. Next, the % nucleotide identity of each pair of orthologs was calculated and based on these % identities for all orthologs within each genome pair, ANI and AND were calculated for all pairs of genomes within the given bacterial species. After calculating AND values, a custom Perl script was used to generate strain dissimilarity matrices, and the trees were generated based on these matrices.

### Placing gene loss events on branches of the phylogenetic trees

In order to re-create the history of gene loss, we used simple parsimony. If a certain gene appears to be lost in two strains, A and B, sharing common ancestor *A*_A,B_, then we predict that it was lost once on the branch leading to the ancestor strain *A*_A,B_. If a certain gene appears to be lost in two strains, that do not share a common ancestor, than we can predict it was lost independently twice through the history of the bacterial species on the branches leading to these strains. This logic allowed us to calculate how many times each near core pangene was likely lost, and to calculate how many genes were likely lost along each branch of each phylogenetic tree.

### Conservation analysis

All completely sequenced and annotated bacterial genomes were downloaded from the NCBI^46^. Bacteria with genomic sequences marked as “draft” as well as data of phages that were sequenced alongside their bacterial hosts were removed from further analysis.

Representative protein sequences of each pangene within a species’ pangenome were compared to the protein sequence data of each bacterium in the NCBI database using FASTA^30^. If the query protein sequence identified a hit within a target bacterium above the threshold of 40% normalized identity, it was considered as present within that bacterium. Normalized identity (NI) was defined as following:


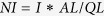


Where **I** = pairwise identity of the compared sequences as identified by FASTA (%), **AL** = sequence alignment length and **QL** = query sequence length.

The NCBI taxonomy database^51^ was used to identify the species-level classification of each strain present in the complete bacterial database, resulting in total of 1496 bacterial species identified. If a pangene was found in at least one representative of a certain species, it was marked as present in that given species. In this manner for each pangene we calculated the number of species it was found in.

### dN/dS calculations

To calculate dN/dS for each pangene, individual gene sequences within each pangene were aligned using MACSE^52^ with default settings and genetic code 11 (The Bacterial, Archaeal and Plant Plastid Code). Next, the PAML (Phylogenetic Analysis by Maximum Likelihood) CodeML program^31^ was used to calculate dN/dS for each pangene, based on the multiple sequence alignment generated usng MACSE. A single dN/dS value was calculated for each species (using the model = 0 setting).

For dN/dS calculation, CodeML^31^ requires a phylogenetic tree of the input sequences. The phylogenetic trees we generated for each species represent all strains of a species and could therefore not be used to calculate dN/dS of near core pangenes that are absent from some strains. To generate trees that contain only those strains in which each near core gene is present, we removed the strains from which the pangene was absent from the dissimilarity matrix used to construct the original trees and reconstructed trees based on these trimmed down matrices.

To avoid possible computational biases due to inclusion of gene sequences with a low number of variable sites, only pangenes with 0.0001 <dN/dS < = 2 and “tree length for dS” ≥0.001 were further considered.

The number of core, single loss and multiple loss pangenes of the investigated species used for statistical analysis after filtering is listed in Table S8. In two bacterial species: MTBC and *Y. pestis*, that we previously demonstrated to be extremely non-diverged in their gene sequences^29^ we were left with insufficient number of pangenes to carry out reliable dN/dS analysis. Therefore, these species were excluded completely from the dN/dS analysis.

### Analysis of codon usage bias

To analyze differences in codon bias among the ‘core’, ‘single loss’ and ‘multiple loss’ pangenes, we used the ENCprime program^53^ with default settings. After calculating ENC’ for each individual gene within given pangene, the average ENC’ value per pangene was calculated. If for a certain pangene the standard deviation of ENC’ values between individual genes was higher than 10%, the pangene was excluded from further analysis. In addition, only data from pangenes in which all genes were at least 100 codons long were included in the analysis.

### Calculating dS along each branch of the species’ phylogenetic trees

The PAML CodeML program^31^ was applied to the ‘core’ pangenes of the investigated bacterial species aligned by MACSE^52^, to evaluate dS values on each branch of each species’ phylogenetic tree. CodeML was run using model = 1 (a branch free model that estimates dS, dN and dN/dS values per branch). The estimation of the dS values was done separately for each ‘core’ pangene. Next, a custom Perl script was used to calculate the average dS (*a*dS) value for each phylogenetic tree branch, across all examined core genes. In order not to rely on gene sequences with very low numbers of variable sites, we excluded genes with dS = 0 or dN/dS was >2, along a certain branch from calculations of *a*dS on that branch.

### Functional analysis of the “core” and “near core” pangenes

Investigated ‘core’ and ‘near core pangenes were queried against the latest COG database downloaded from the NCBI using FASTA^30^. A certain COG classification was assigned to a pangene, if the representative query gene from that pangene identified a hit within COG’s sequences with at least 70% identity, across at least 2/3 of the query. For each species studied, we then calculated what percentage of ‘near core’ and ‘core’ pangenes received each possible COG classification. Finally, a two-tailed, paired Mann-Whitney test was used to examine for each COG classification whether, across the 15 species studied, there was a significant difference in the percentage of ‘core’ and ‘near core pangenes receiving that COG classification. The results of this statistical test are given in Table S6.

## Additional Information

**How to cite this article**: Bolotin, E. and Hershberg, R. Bacterial intra-species gene loss occurs in a largely clocklike manner mostly within a pool of less conserved and constrained genes. *Sci. Rep.* **6**, 35168; doi: 10.1038/srep35168 (2016).

## Supplementary Material

Supplementary Information

## Figures and Tables

**Table 1:** Pangenome data summary of the analyzed species.

Species Name	Analyzed genomes	Core pangenes	Near core pangenes	Single loss pangenes	Multiple loss pangenes
*Bacillus cereus*	13	3165	630	512	118
*Clostridium botulinum*	10	2270	591	479	112
*Corynebacterium diphtheriae*	13	1630	145	119	26
*Campylobacter jejuni*	13	1012	360	322	38
*Corynebacterium pseudotuberculosis*	15	1471	415	298	117
*Eschirechia coli-Shigella*	60	1456	1625	639	986
*Francisella tularensis*	12	1010	186	130	56
*Haemophilus influenzae*	10	1026	354	300	54
*Listeria monocytogenes*	26	2174	357	296	61
*Mycobacterium Tuberculosis* Complex	20	2665	746	527	219
*Neisseria meningitidis*	14	1270	199	133	66
*Pseudomonas aeruginosa*	13	4332	779	652	127
*Streptococcus pneumoniae*	21	1202	292	188	104
*Streptococcus suis*	17	1056	473	357	116
*Yersinia pestis*	12	2396	639	507	132
