# Supplementary material for: Bacterial intra-species gene loss occurs in a largely clocklike manner mostly within a pool of less conserved and constrained genes
Source: Sci Rep. 2016 Oct 13;6:35168. doi: 10.1038/srep35168 (PMC5062063; doi:10.1038/srep35168)
Supplement: Supplementary Information [file srep35168-s1.pdf]

Bacterial intra-species gene loss occurs in a largely clocklike manner mostly within a pool of less conserved and constrained genes

Authors: Evgeni Bolotin and Ruth Hershberg

B. cereus

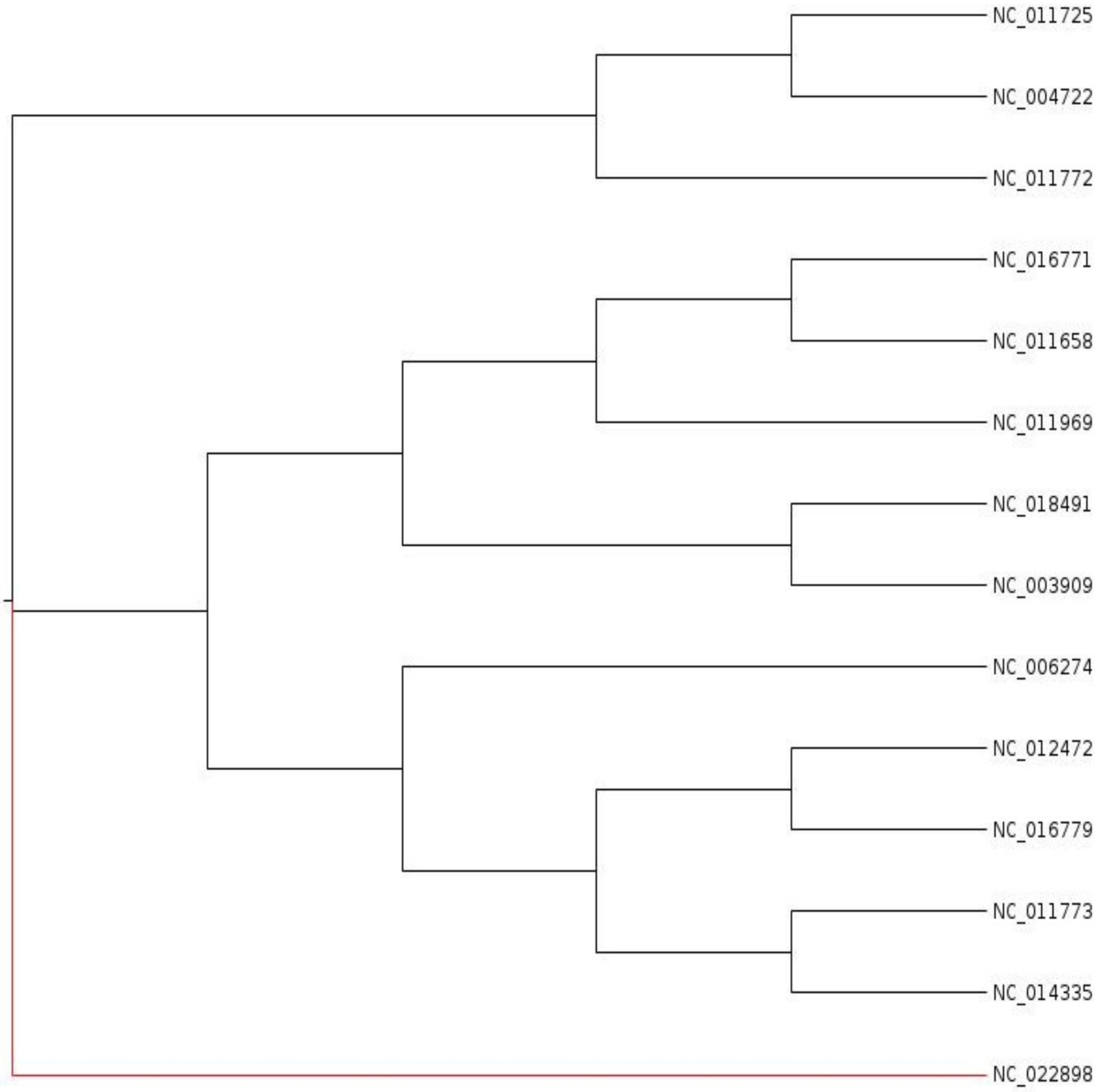

NC_011725
NC_004722
NC_011772
NC_016771
NC_011658
NC_011969
NC_018491
NC_003909
NC_006274
NC_012472
NC_016779
NC_011773
NC_014335
NC_022898

C. botulinum

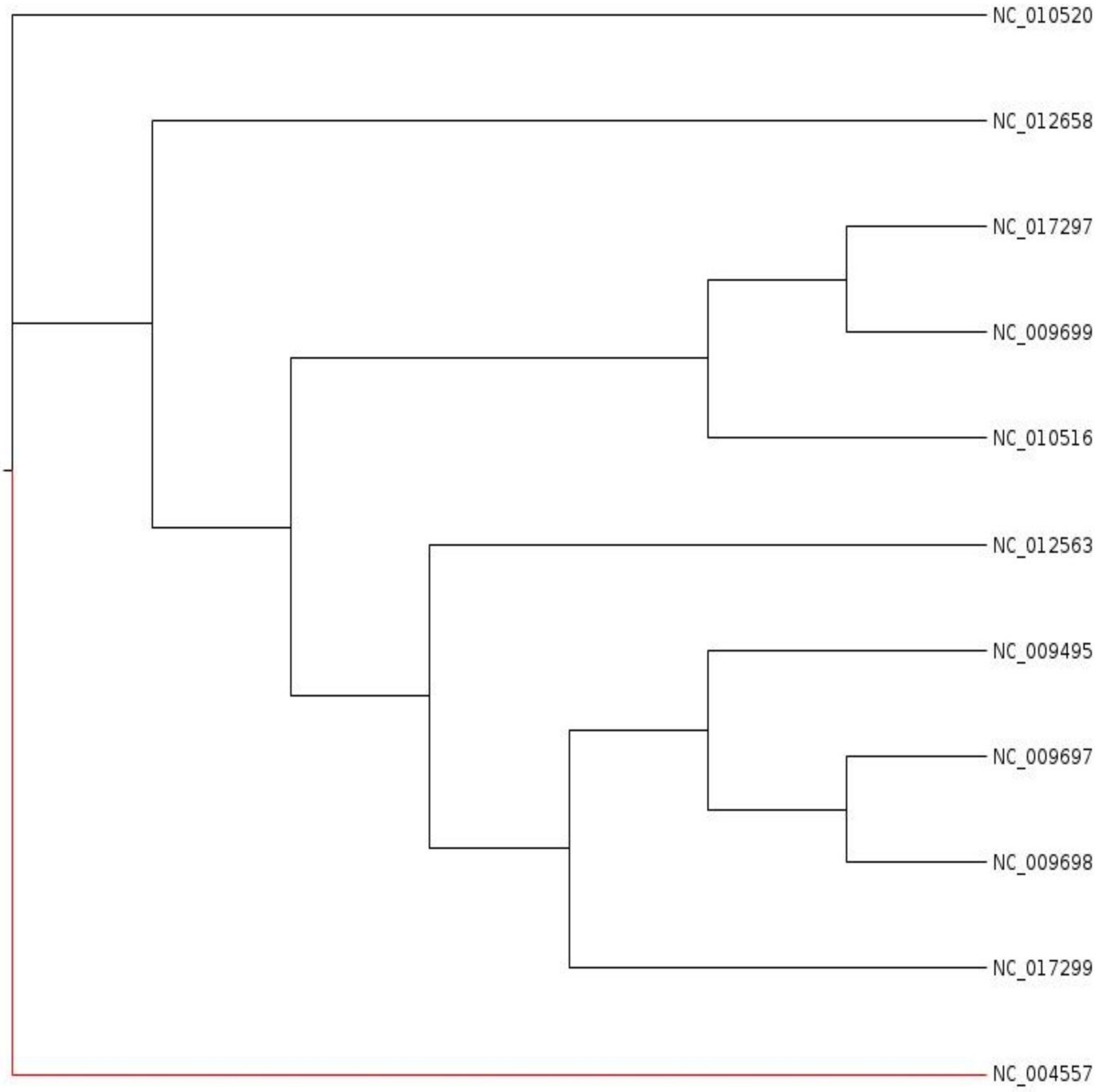

NC_010520
NC_012658
NC_017297
NC_009699
NC_010516
NC_012563
NC_009495
NC_009697
NC_009698
NC_017299
NC_004557

C. diphtheriae

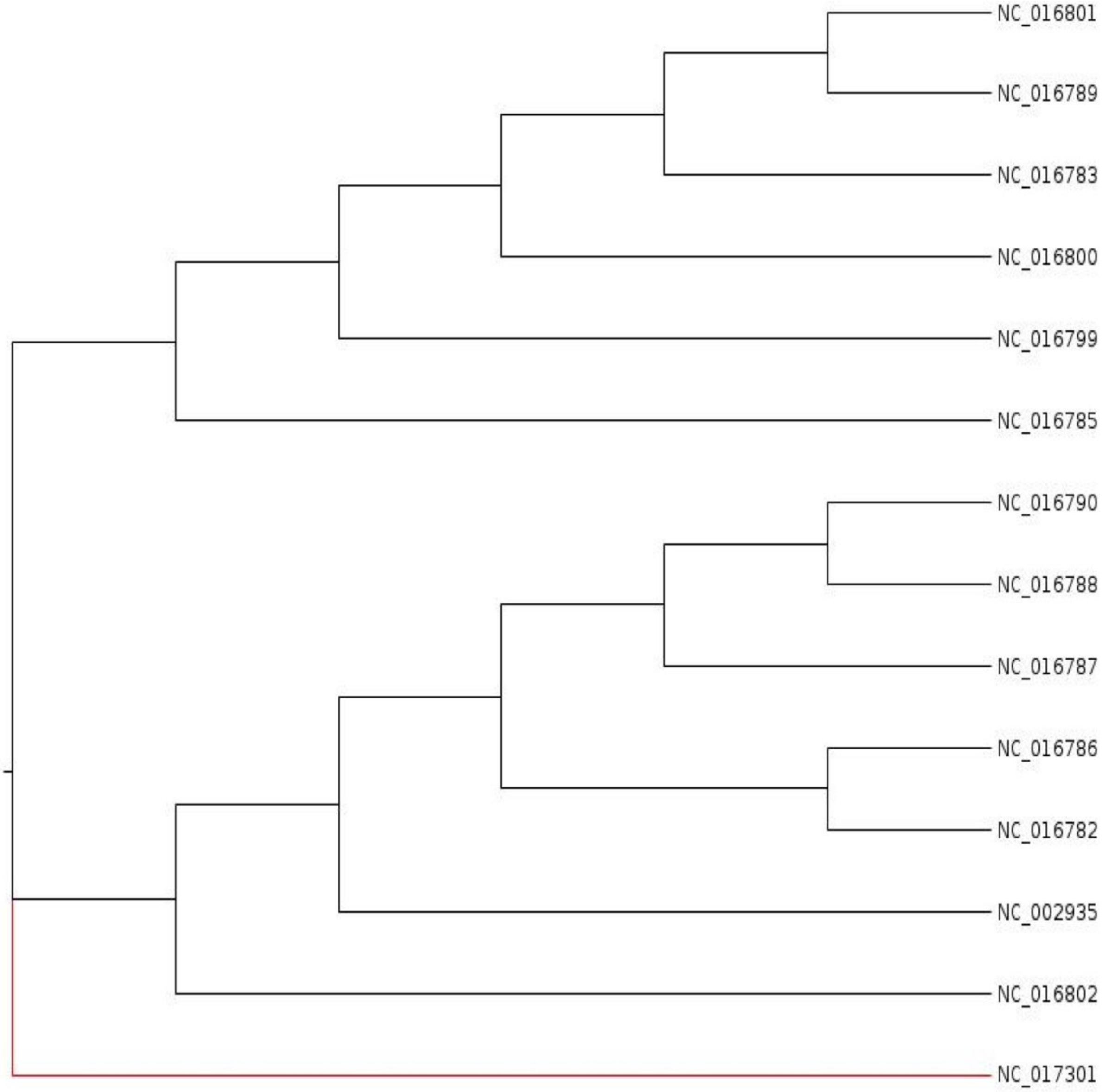

C. jejuni

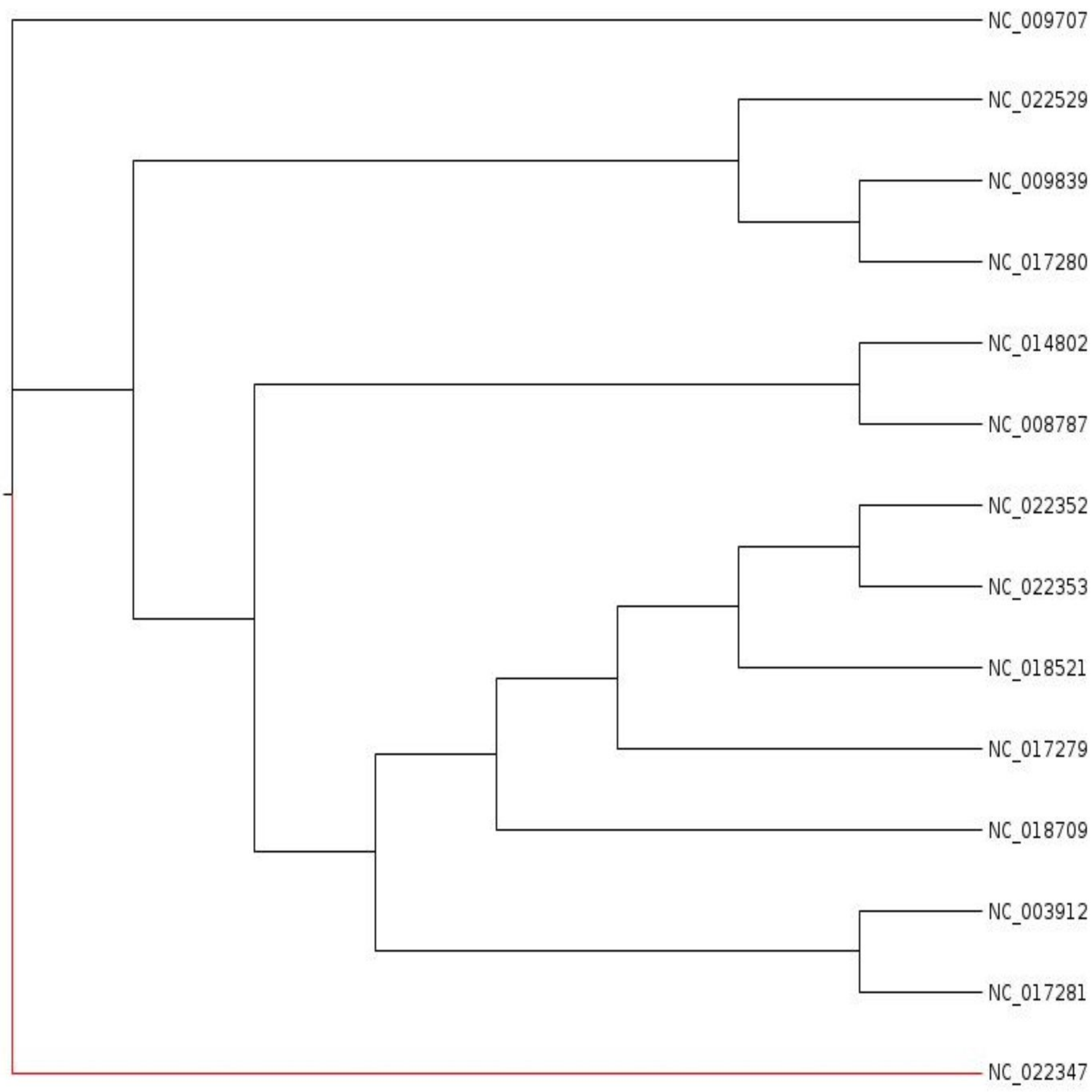

NC_009707
NC_022529
NC_009839
NC_017280
NC_014802
NC_008787
NC_022352
NC_022353
NC_018521
NC_017279
NC_018709
NC_003912
NC_017281
NC_022347

C. pseudotuberculosis

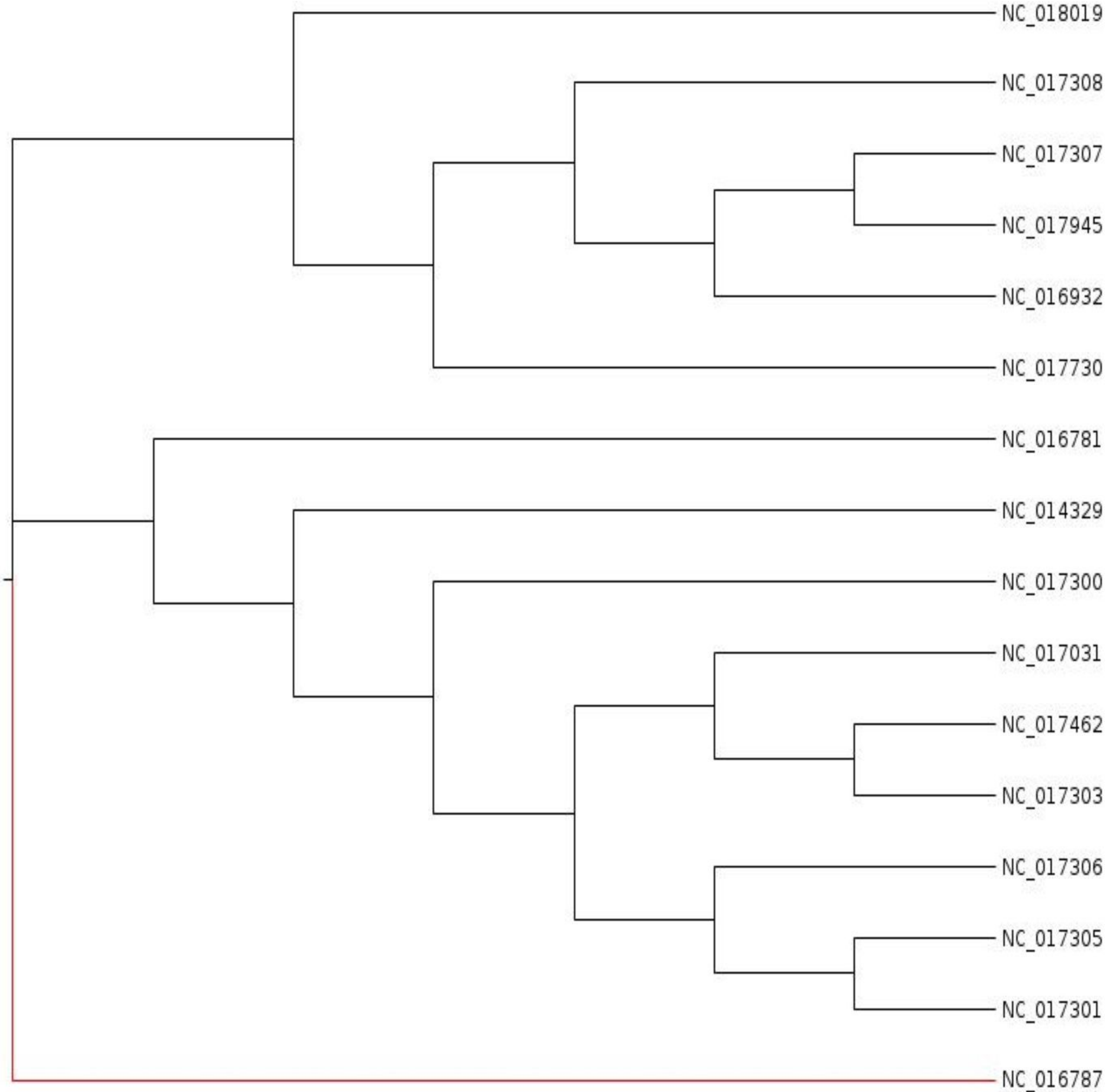

E. coli - Shigella

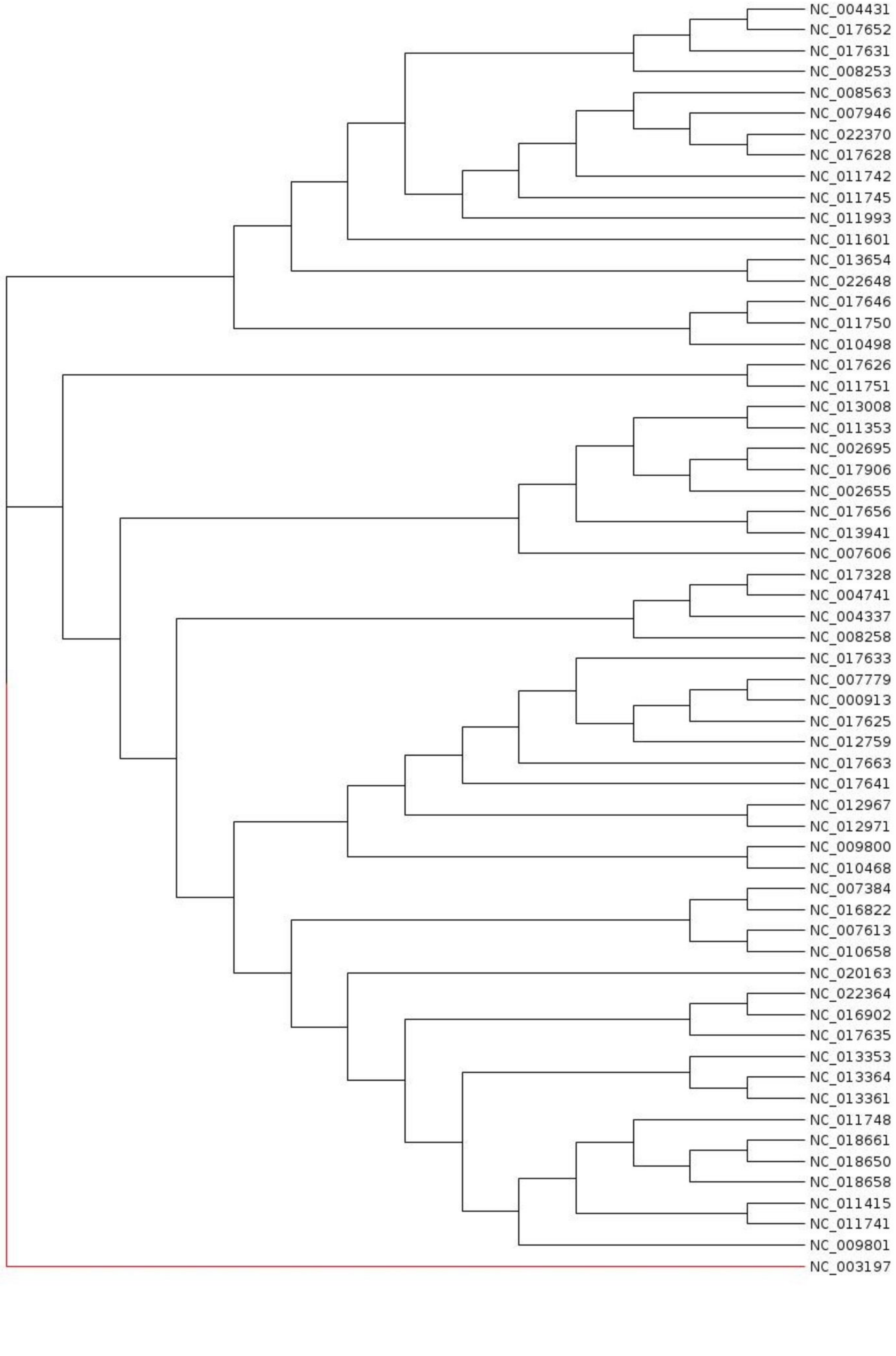

F. tularensis

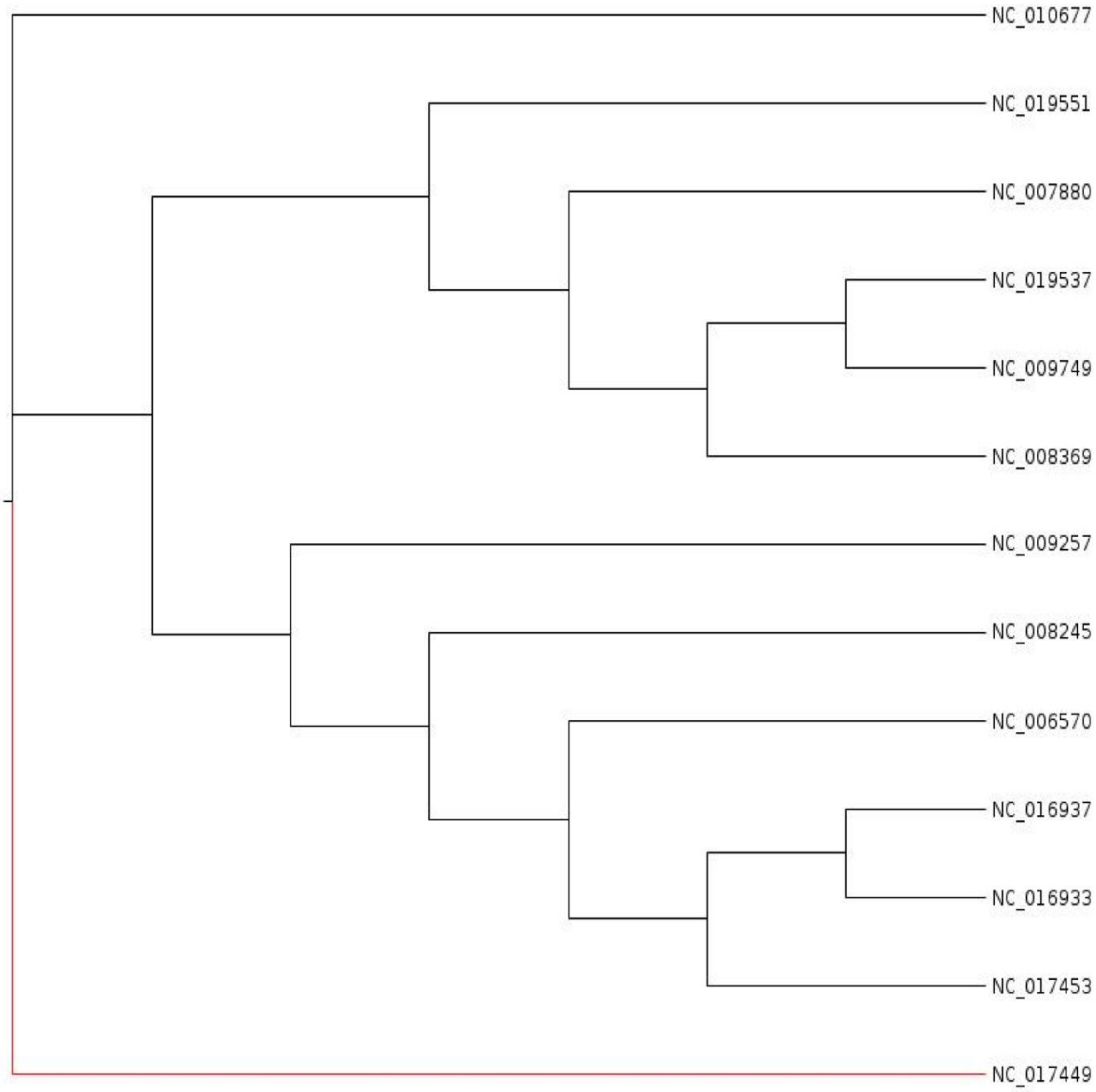

H. influenzae

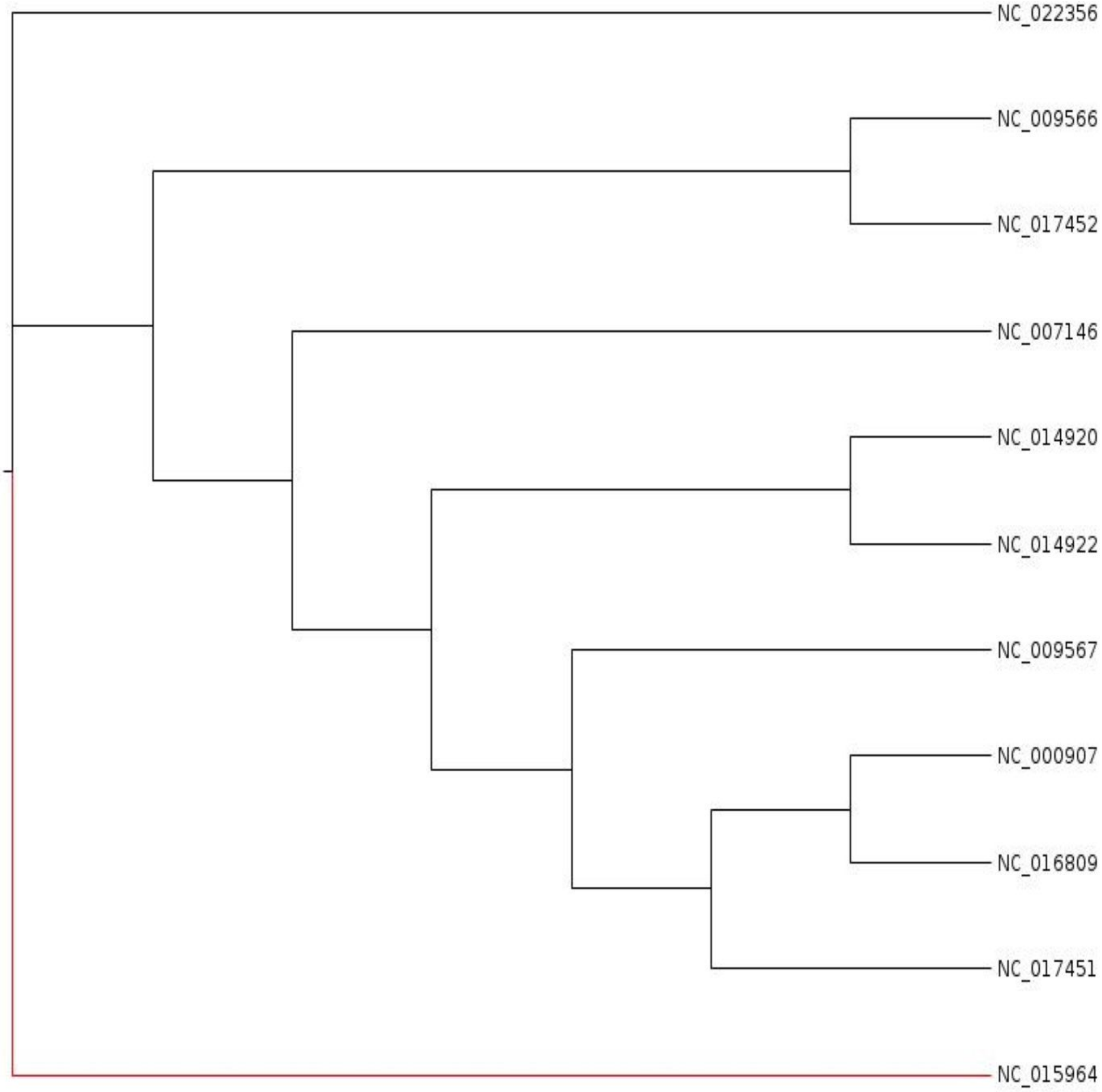

L. monocytogenes

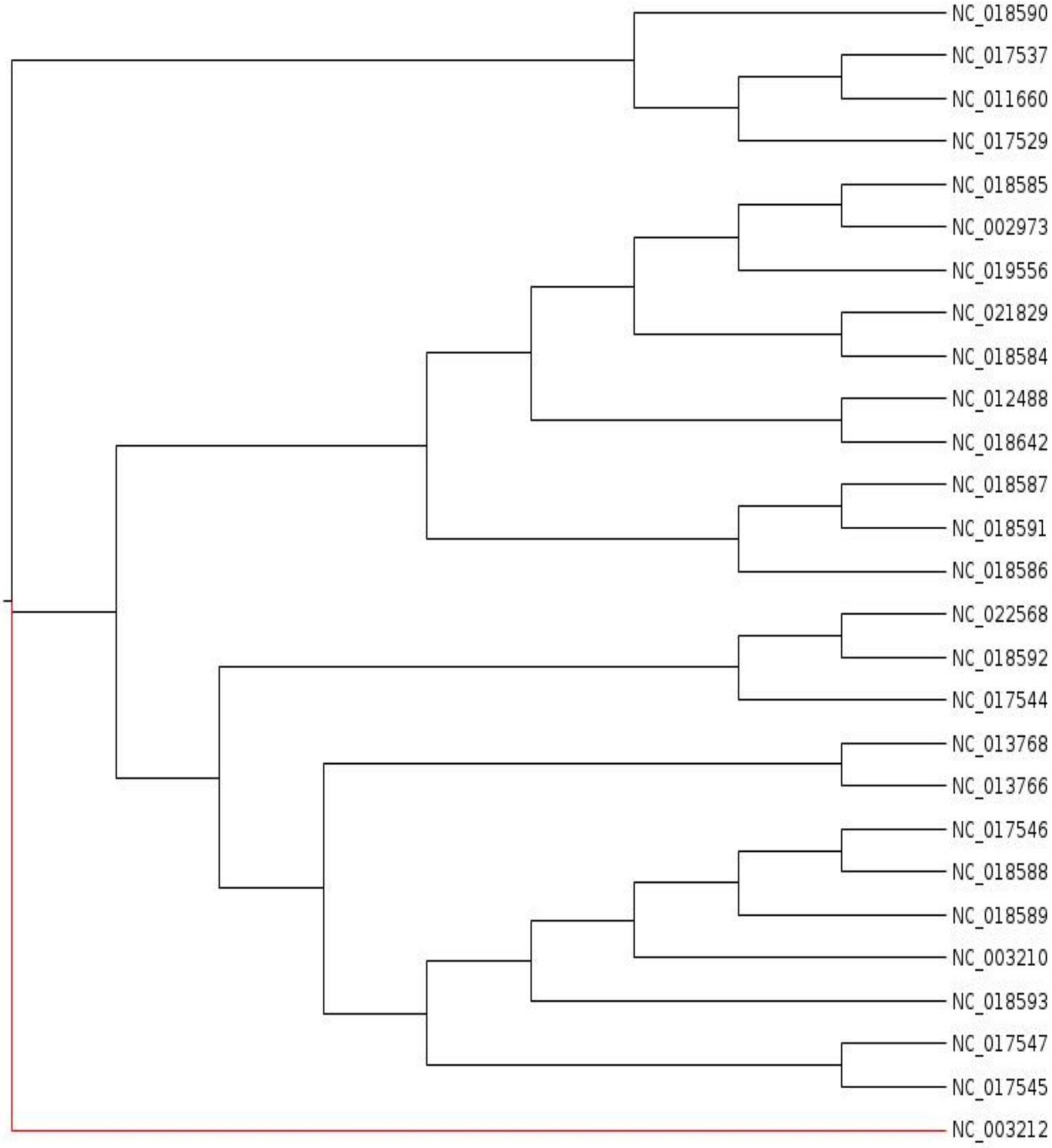

MTBC

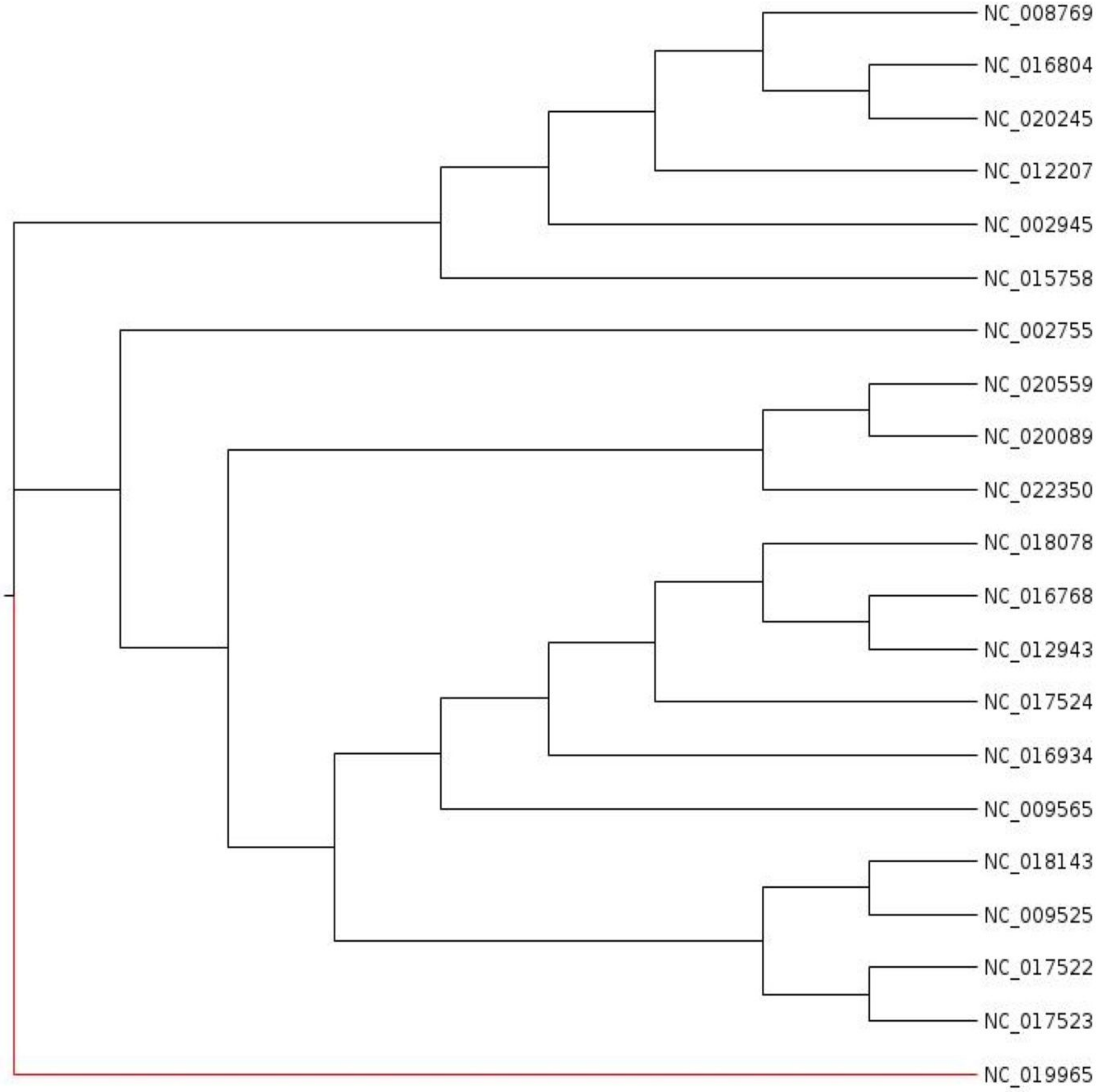
NC_008769
NC_016804
NC_020245
NC_012207
NC_002945
NC_015758
NC_002755
NC_020559
NC_020089
NC_022350
NC_018078
NC_016768
NC_012943
NC_017524
NC_016934
NC_009565
NC_018143
NC_009525
NC_017522
NC_017523
NC_019965

N. meningitidis

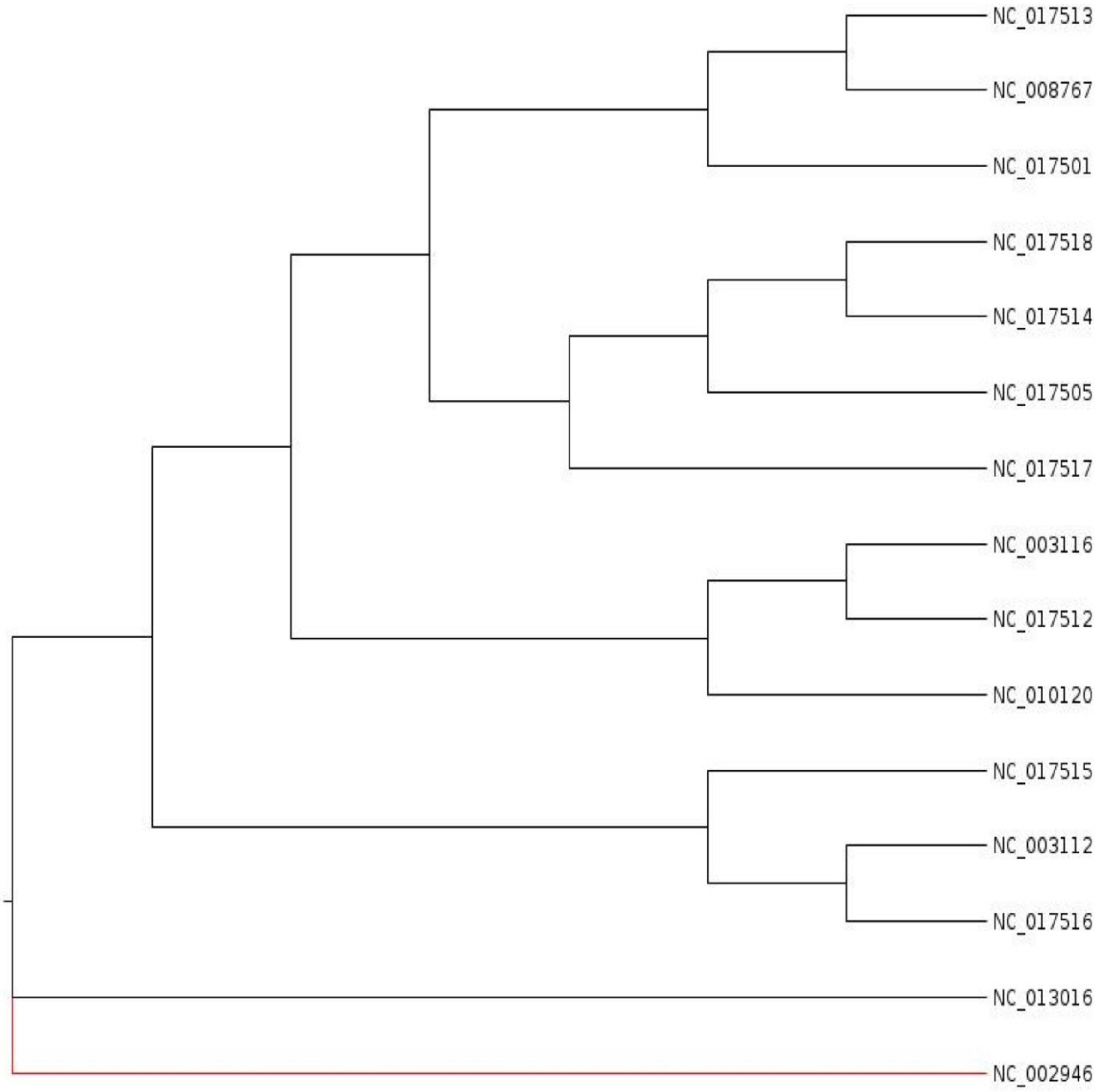

P. aeruginosa

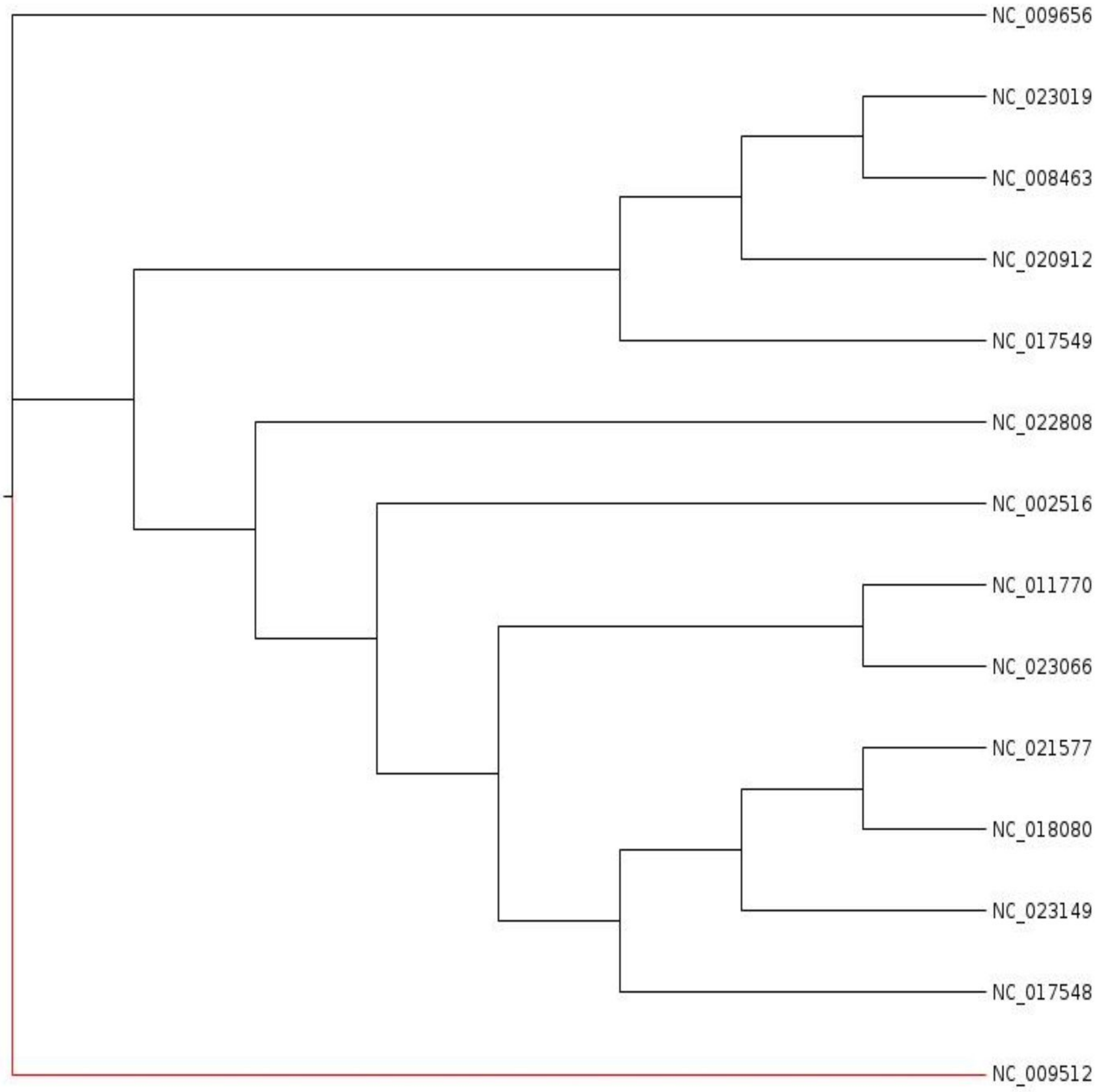

S. pneumoniae

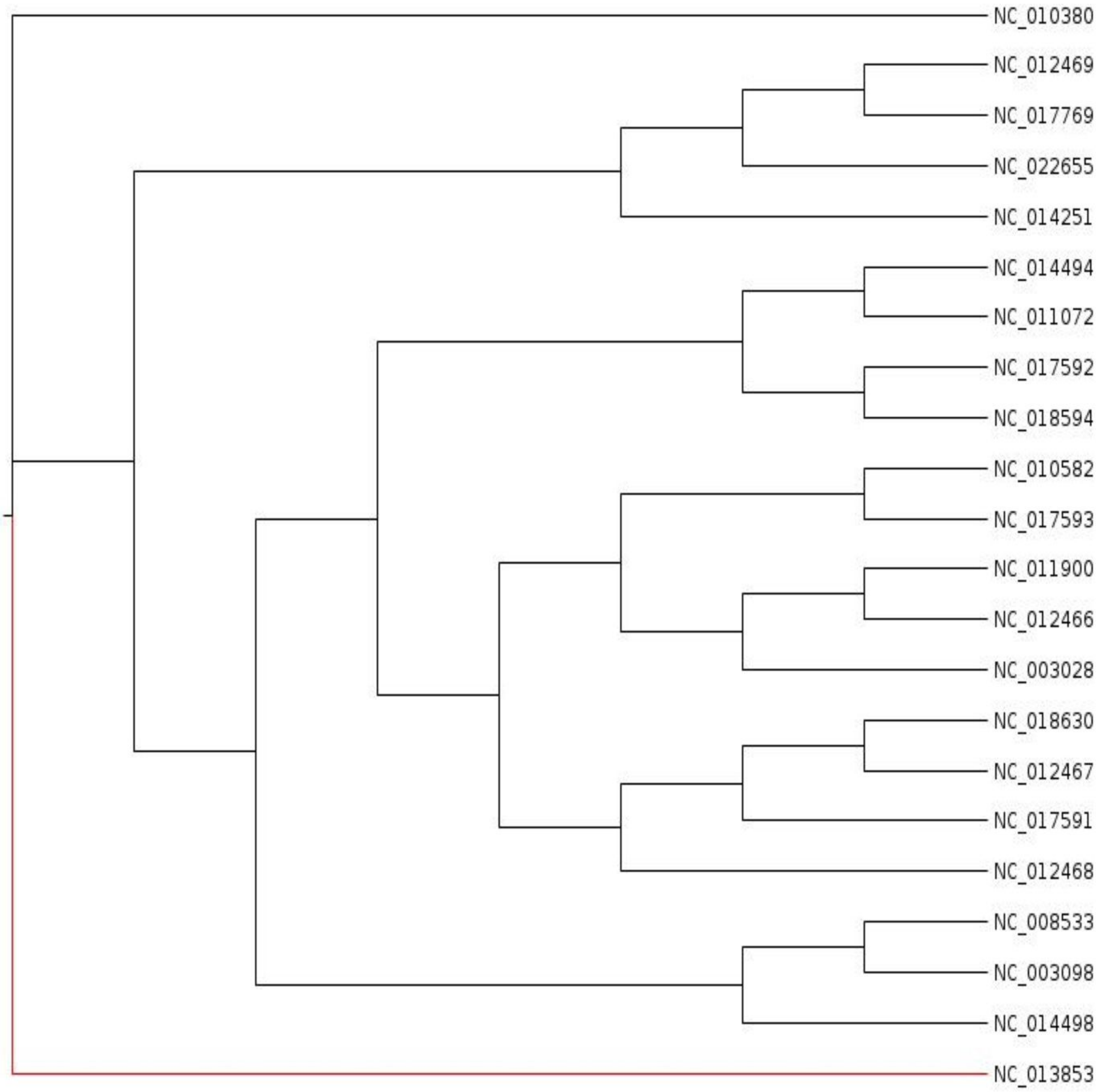

S. suis

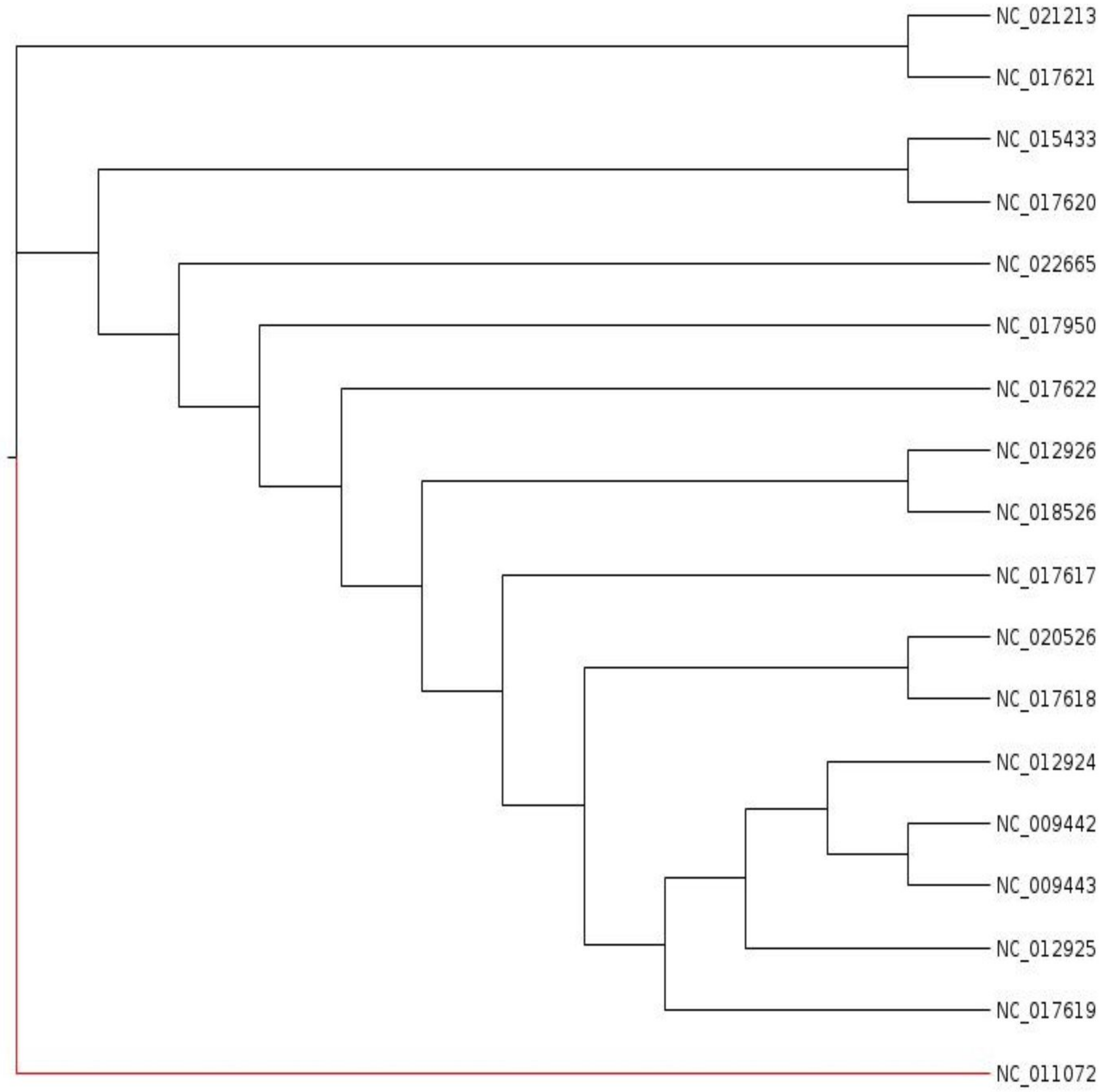
NC_021213
NC_017621
NC_015433
NC_017620
NC_022665
NC_017950
NC_017622
NC_012926
NC_018526
NC_017617
NC_020526
NC_017618
NC_012924
NC_009442
NC_009443
NC_012925
NC_017619
NC_011072

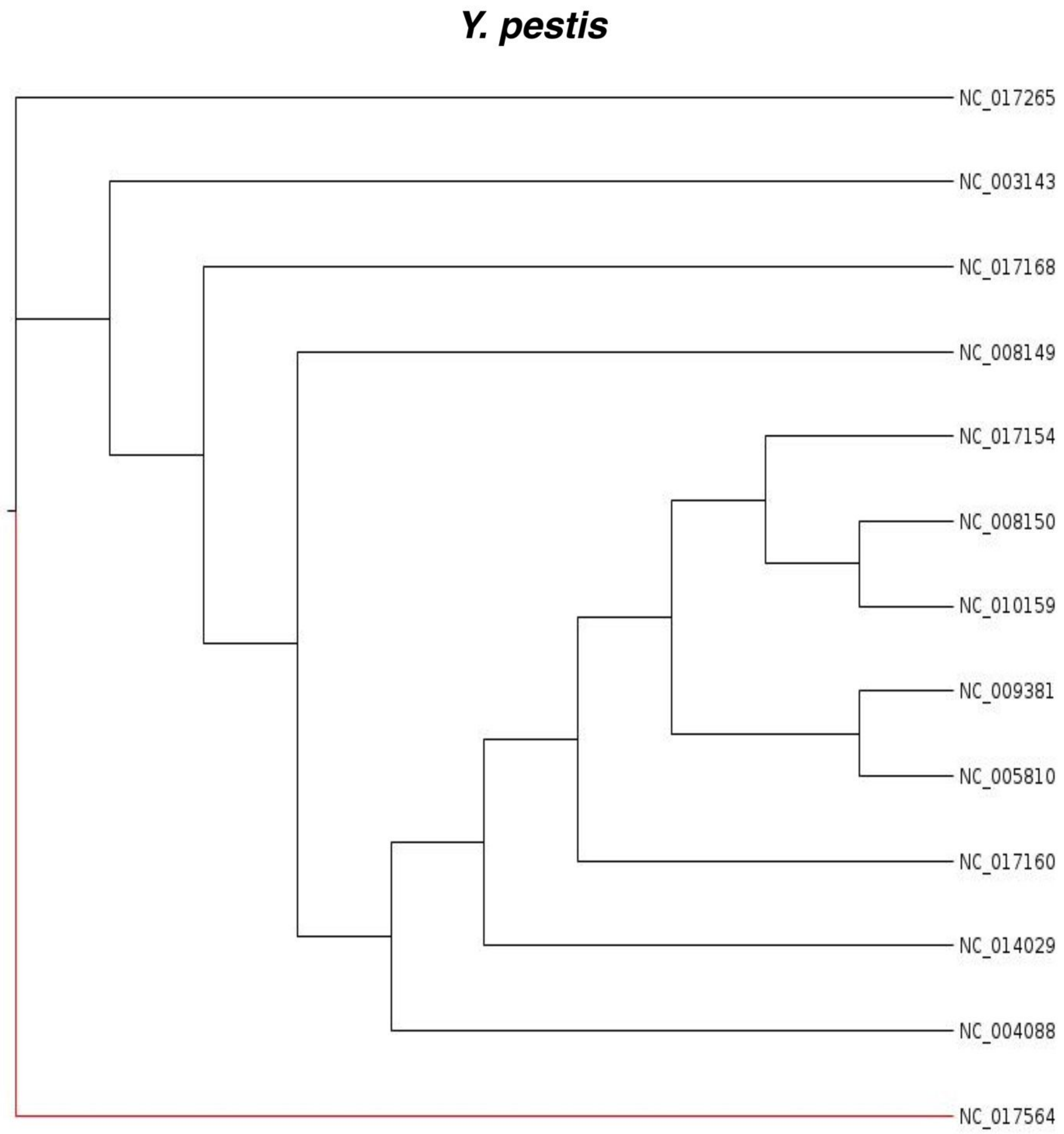

Figure S1. **Phylogentic relations between the strains in the investigated bacterial species/groups based on the average sequence dissimilarity between the "core" non-recombining genes in the NCBI-annotated pangenomes.** The branch leading to an outgroup strain is shown in red.

Table S1. NCBI accession numbers and names of the investigated bacterial strains. The names here are given as they appear in the NCBI annotation files.

Bacilllus cereus	
**Accession number**	**Strain Name**
NC_003909	Bacillus cereus ATCC 10987
NC_004722	Bacillus cereus ATCC 14579
NC_006274	Bacillus cereus E33L
NC_011658	Bacillus cereus AH187
NC_011725	Bacillus cereus B4264
NC_011772	Bacillus cereus G9842
NC_011773	Bacillus cereus AH820
NC_011969	Bacillus cereus Q1
NC_012472	Bacillus cereus 03BB102
NC_014335	Bacillus cereus biovar anthracis str. CI
NC_016771	Bacillus cereus NC7401
NC_016779	Bacillus cereus F837/76
NC_018491	Bacillus cereus FRI-35
Campylobacter jejuni	
**Accession number**	**Strain Name**
NC_003912	Campylobacter jejuni RM1221
NC_008787	Campylobacter jejuni subsp. jejuni 81-176
NC_009707	Campylobacter jejuni subsp. doylei 269.97
NC_009839	Campylobacter jejuni subsp. jejuni 81116
NC_014802	Campylobacter jejuni subsp. jejuni ICDCCJ07001
NC_017279	Campylobacter jejuni subsp. jejuni IA3902
NC_017280	Campylobacter jejuni subsp. jejuni M1
NC_017281	Campylobacter jejuni subsp. jejuni S3
NC_018521	Campylobacter jejuni subsp. jejuni NCTC 11168-BN148
NC_018709	Campylobacter jejuni subsp. jejuni PT14
NC_022352	Campylobacter jejuni subsp. jejuni 00-2426
NC_022353	Campylobacter jejuni subsp. jejuni 00-2544
NC_022529	Campylobacter jejuni 4031

Clostridium botulinum	
**Accession number**	**Strain Name**
NC_009495	Clostridium botulinum A str. ATCC 3502
NC_009697	Clostridium botulinum A str. ATCC 19397
NC_009698	Clostridium botulinum A str. Hall
NC_009699	Clostridium botulinum F str. Langeland
NC_010516	Clostridium botulinum B1 str. Okra
NC_010520	Clostridium botulinum A3 str. Loch Maree
NC_012563	Clostridium botulinum A2 str. Kyoto
NC_012658	Clostridium botulinum Ba4 str. 657
NC_017297	Clostridium botulinum F str. 230613
NC_017299	Clostridium botulinum H04402 065
Corynebacterium diphtheriae	
**Accession number**	**Strain Name**
NC_002935	Corynebacterium diphtheriae NCTC 13129
NC_016782	Corynebacterium diphtheriae 241
NC_016783	Corynebacterium diphtheriae INCA 402
NC_016785	Corynebacterium diphtheriae CDCE 8392
NC_016786	Corynebacterium diphtheriae HC01
NC_016787	Corynebacterium diphtheriae HC03
NC_016788	Corynebacterium diphtheriae HC04
NC_016789	Corynebacterium diphtheriae PW8
NC_016790	Corynebacterium diphtheriae VA01
NC_016799	Corynebacterium diphtheriae 31A
NC_016800	Corynebacterium diphtheriae BH8
NC_016801	Corynebacterium diphtheriae C7 (beta)
NC_016802	Corynebacterium diphtheriae HC02
Corynebacterium pseudotuberculosis	
**Accession number**	**Strain Name**
NC_014329	Corynebacterium pseudotuberculosis FRC41
NC_016781	Corynebacterium pseudotuberculosis 3/99-5
NC_016932	Corynebacterium pseudotuberculosis 316

NC_017031	Corynebacterium pseudotuberculosis P54B96
NC_017300	Corynebacterium pseudotuberculosis 1002
NC_017301	Corynebacterium pseudotuberculosis C231
NC_017303	Corynebacterium pseudotuberculosis I19
NC_017305	Corynebacterium pseudotuberculosis PAT10
NC_017306	Corynebacterium pseudotuberculosis 42/02-A
NC_017307	Corynebacterium pseudotuberculosis CIP 52.97
NC_017308	Corynebacterium pseudotuberculosis 1/06-A
NC_017462	Corynebacterium pseudotuberculosis 267
NC_017730	Corynebacterium pseudotuberculosis 31
NC_017945	Corynebacterium pseudotuberculosis 258
NC_018019	Corynebacterium pseudotuberculosis Cp162
Escherichia coli-Shigella	
**Accession number**	**Strain Name**
NC_000913	Escherichia coli str. K-12 substr. MG1655
NC_002655	Escherichia coli O157:H7 str. EDL933
NC_002695	Escherichia coli O157:H7 str. Sakai
NC_004431	Escherichia coli CFT073
NC_007779	Escherichia coli str. K-12 substr. W3110
NC_007946	Escherichia coli UTI89
NC_008253	Escherichia coli 536
NC_008563	Escherichia coli APEC O1
NC_009800	Escherichia coli HS
NC_009801	Escherichia coli E24377A
NC_010468	Escherichia coli ATCC 8739
NC_010498	Escherichia coli SMS-3-5
NC_011353	Escherichia coli O157:H7 str. EC4115
NC_011415	Escherichia coli SE11
NC_011601	Escherichia coli O127:H6 str. E2348/69
NC_011741	Escherichia coli IAI1
NC_011742	Escherichia coli S88
NC_011745	Escherichia coli ED1a

NC_011748	Escherichia coli 55989
NC_011750	Escherichia coli IAI39
NC_011751	Escherichia coli UMN026
NC_011993	Escherichia coli LF82
NC_012759	Escherichia coli BW2952
NC_012967	Escherichia coli B str. REL606
NC_012971	Escherichia coli BL21(DE3)
NC_013008	Escherichia coli O157:H7 str. TW14359
NC_013353	Escherichia coli O103:H2 str. 12009
NC_013361	Escherichia coli O26:H11 str. 11368
NC_013364	Escherichia coli O111:H- str. 11128
NC_013654	Escherichia coli SE15
NC_013941	Escherichia coli O55:H7 str. CB9615
NC_016902	Escherichia coli KO11FL
NC_017625	Escherichia coli DH1
NC_017626	Escherichia coli 042
NC_017628	Escherichia coli IHE3034
NC_017631	Escherichia coli ABU 83972
NC_017633	Escherichia coli ETEC H10407
NC_017635	Escherichia coli W
NC_017641	Escherichia coli UMNK88
NC_017646	Escherichia coli O7:K1 str. CE10
NC_017652	Escherichia coli str. 'clone D i14'
NC_017656	Escherichia coli O55:H7 str. RM12579
NC_017663	Escherichia coli P12b
NC_017906	Escherichia coli Xuzhou21
NC_018650	Escherichia coli O104:H4 str. 2009EL-2050
NC_018658	Escherichia coli O104:H4 str. 2011C-3493
NC_018661	Escherichia coli O104:H4 str. 2009EL-2071
NC_020163	Escherichia coli APEC O78
NC_022364	Escherichia coli LY180
NC_022370	Escherichia coli PMV-1

NC_022648	Escherichia coli JJ1886
NC_004337	Shigella flexneri 2a str. 301
NC_004741	Shigella flexneri 2a str. 2457T
NC_007384	Shigella sonnei Ss046
NC_007606	Shigella dysenteriae Sd197
NC_007613	Shigella boydii Sb227
NC_008258	Shigella flexneri 5 str. 8401
NC_010658	Shigella boydii CDC 3083-94
NC_016822	Shigella sonnei 53G
NC_017328	Shigella flexneri 2002017
Francisella tularensis	
**Accession number**	**Strain Name**
NC_006570	Francisella tularensis subsp. tularensis SCHU S4
NC_007880	Francisella tularensis subsp. holarctica LVS
NC_008245	Francisella tularensis subsp. tularensis FSC198
NC_008369	Francisella tularensis subsp. holarctica OSU18
NC_009257	Francisella tularensis subsp. tularensis WY96-3418
NC_009749	Francisella tularensis subsp. holarctica FTNF002-00
NC_010677	Francisella tularensis subsp. mediasiatica FSC147
NC_016933	Francisella tularensis TIGB03
NC_016937	Francisella tularensis subsp. tularensis TI0902
NC_017453	Francisella tularensis subsp. tularensis NE061598
NC_019537	Francisella tularensis subsp. holarctica F92
NC_019551	Francisella tularensis subsp. holarctica FSC200
Haemophilus influenzae	
**Accession number**	**Strain Name**
NC_000907	Haemophilus influenzae Rd KW20
NC_007146	Haemophilus influenzae 86-028NP
NC_009566	Haemophilus influenzae PittEE
NC_009567	Haemophilus influenzae PittGG
NC_014920	Haemophilus influenzae F3031
NC_014922	Haemophilus influenzae F3047

NC_016809	Haemophilus influenzae 10810
NC_017451	Haemophilus influenzae R2866
NC_017452	Haemophilus influenzae R2846
NC_022356	Haemophilus influenzae KR494
Listeria monocytogenes	
**Accession number**	**Strain Name**
NC_002973	Listeria monocytogenes serotype 4b str. F2365
NC_003210	Listeria monocytogenes EGD-e
NC_011660	Listeria monocytogenes HCC23
NC_012488	Listeria monocytogenes Clip81459
NC_013766	Listeria monocytogenes 08-5578
NC_013768	Listeria monocytogenes 08-5923
NC_017529	Listeria monocytogenes L99
NC_017537	Listeria monocytogenes M7
NC_017544	Listeria monocytogenes 10403S
NC_017545	Listeria monocytogenes J0161
NC_017546	Listeria monocytogenes FSL R2-561
NC_017547	Listeria monocytogenes Finland 1998
NC_018584	Listeria monocytogenes ATCC 19117
NC_018585	Listeria monocytogenes SLCC2378
NC_018586	Listeria monocytogenes SLCC2540
NC_018587	Listeria monocytogenes SLCC2755
NC_018588	Listeria monocytogenes SLCC2372
NC_018589	Listeria monocytogenes SLCC2479
NC_018590	Listeria monocytogenes SLCC2376
NC_018591	Listeria monocytogenes serotype 7 str. SLCC2482
NC_018592	Listeria monocytogenes SLCC5850
NC_018593	Listeria monocytogenes SLCC7179
NC_018642	Listeria monocytogenes L312
NC_019556	Listeria monocytogenes serotype 4b str. LL195
NC_021829	Listeria monocytogenes J1816
NC_022568	Listeria monocytogenes EGD

Mycobacterium tuberculosis **Complex**	
**Accession number**	**Strain Name**
NC_002755	Mycobacterium tuberculosis CDC1551
NC_002945	Mycobacterium bovis AF2122/97
NC_008769	Mycobacterium bovis BCG str. Pasteur 1173P2
NC_009525	Mycobacterium tuberculosis H37Ra
NC_009565	Mycobacterium tuberculosis F11
NC_012207	Mycobacterium bovis BCG str. Tokyo 172
NC_012943	Mycobacterium tuberculosis KZN 1435
NC_015758	Mycobacterium africanum GM041182
NC_016768	Mycobacterium tuberculosis KZN 4207
NC_016804	Mycobacterium bovis BCG str. Mexico
NC_016934	Mycobacterium tuberculosis UT205
NC_017522	Mycobacterium tuberculosis CCDC5180
NC_017523	Mycobacterium tuberculosis CCDC5079
NC_017524	Mycobacterium tuberculosis CTRI-2
NC_018078	Mycobacterium tuberculosis KZN 605
NC_018143	Mycobacterium tuberculosis H37Rv
NC_020089	Mycobacterium tuberculosis 7199-99
NC_020245	Mycobacterium bovis BCG str. Korea 1168P
NC_020559	Mycobacterium tuberculosis str. Erdman = ATCC 35801
NC_022350	Mycobacterium tuberculosis str. Haarlem
Neisseria meningitidis	
**Accession number**	**Strain Name**
NC_003112	Neisseria meningitidis MC58
NC_003116	Neisseria meningitidis Z2491
NC_008767	Neisseria meningitidis FAM18
NC_010120	Neisseria meningitidis 053442
NC_013016	Neisseria meningitidis alpha14
NC_017501	Neisseria meningitidis 8013
NC_017505	Neisseria meningitidis alpha710
NC_017512	Neisseria meningitidis WUE 2594

NC_017513	Neisseria meningitidis G2136
NC_017514	Neisseria meningitidis M01-240149
NC_017515	Neisseria meningitidis M04-240196
NC_017516	Neisseria meningitidis H44/76
NC_017517	Neisseria meningitidis M01-240355
NC_017518	Neisseria meningitidis NZ-05/33
Pseudomonas aeruginosa	
**Accession number**	**Strain Name**
NC_002516	Pseudomonas aeruginosa PAO1
NC_008463	Pseudomonas aeruginosa UCBPP-PA14
NC_009656	Pseudomonas aeruginosa PA7
NC_011770	Pseudomonas aeruginosa LESB58
NC_017548	Pseudomonas aeruginosa M18
NC_017549	Pseudomonas aeruginosa NCGM2.S1
NC_018080	Pseudomonas aeruginosa DK2
NC_020912	Pseudomonas aeruginosa B136-33
NC_021577	Pseudomonas aeruginosa RP73
NC_022808	Pseudomonas aeruginosa PA1
NC_023019	Pseudomonas aeruginosa MTB-1
NC_023066	Pseudomonas aeruginosa LES431
NC_023149	Pseudomonas aeruginosa SCV20265
Streptococcus pneumoniae	
**Accession number**	**Strain Name**
NC_003028	Streptococcus pneumoniae TIGR4
NC_003098	Streptococcus pneumoniae R6
NC_008533	Streptococcus pneumoniae D39
NC_010380	Streptococcus pneumoniae Hungary19A-6
NC_010582	Streptococcus pneumoniae CGSP14
NC_011072	Streptococcus pneumoniae G54
NC_011900	Streptococcus pneumoniae ATCC 700669
NC_012466	Streptococcus pneumoniae JJA
NC_012467	Streptococcus pneumoniae P1031

NC_012468	Streptococcus pneumoniae 70585
NC_012469	Streptococcus pneumoniae Taiwan19F-14
NC_014251	Streptococcus pneumoniae TCH8431/19A
NC_014494	Streptococcus pneumoniae AP200
NC_014498	Streptococcus pneumoniae 670-6B
NC_017591	Streptococcus pneumoniae INV104
NC_017592	Streptococcus pneumoniae OXC141
NC_017593	Streptococcus pneumoniae INV200
NC_017769	Streptococcus pneumoniae ST556
NC_018594	Streptococcus pneumoniae SPNA45
NC_018630	Streptococcus pneumoniae PNI0373
NC_022655	Streptococcus pneumoniae A026
Streptococcus suis	
**Accession number**	**Strain Name**
NC_009442	Streptococcus suis 05ZYH33
NC_009443	Streptococcus suis 98HAH33
NC_012924	Streptococcus suis SC84
NC_012925	Streptococcus suis P1/7
NC_012926	Streptococcus suis BM407
NC_015433	Streptococcus suis ST3
NC_017617	Streptococcus suis GZ1
NC_017618	Streptococcus suis JS14
NC_017619	Streptococcus suis SS12
NC_017620	Streptococcus suis D9
NC_017621	Streptococcus suis D12
NC_017622	Streptococcus suis A7
NC_017950	Streptococcus suis ST1
NC_018526	Streptococcus suis S735
NC_020526	Streptococcus suis SC070731
NC_021213	Streptococcus suis TL13
NC_022665	Streptococcus suis T15
Yersinia pestis	

Accession number	Strain Name
NC_003143	Yersinia pestis CO92
NC_004088	Yersinia pestis KIM 10
NC_005810	Yersinia pestis biovar Microtus str. 91001
NC_008149	Yersinia pestis Nepal516
NC_008150	Yersinia pestis Antiqua
NC_009381	Yersinia pestis Pestoides F
NC_010159	Yersinia pestis Angola
NC_014029	Yersinia pestis Z176003
NC_017154	Yersinia pestis D106004
NC_017160	Yersinia pestis D182038
NC_017168	Yersinia pestis A1122
NC_017265	Yersinia pestis biovar Medievalis str. Harbin 35

Table S2. Comparison of differences in the evolutionary conservation values between the “core”, “single-loss” and “multiple-loss” pangene groups in the NCBI-annotated pangenomes using one-sided Mann-Whitney-Wilcoxon test

Organism	Compared gene-loss groups	Number of pangenes in each group	U-test W	P-value
B. cereus	“Core” – “Single-loss”	3165, 512	998591	< 2.2E-16
	“Single-loss” – “Multiple-loss”	512, 118	36078	4.90E-04
C. botulinum	“Core” – “Single-loss”	2270, 479	678352	< 2.2E-16
	“Single-loss” – “Multiple-loss”	479, 112	29994	2.56E-02
C. diphtheriae	“Core” – “Single-loss”	1630, 119	130960	8.37E-11
	“Single-loss” – “Multiple-loss”	119, 26	1983.5	1.21E-02
C. jejuni	“Core” – “Single-loss”	1012, 322	190080	3.23E-06
	“Single-loss” – “Multiple-loss”	322, 38	7562.5	8.61E-03
C. pseudotuberculosis*	“Core” – “Single-loss”	1471, 298	275880	8.82E-13
	“Single-loss” – “Multiple-loss”	298, 117	22620	1.17E-06
E. coli-Shigella	“Core” – “Single-loss”	1456, 639	578800	< 2.2E-16
	“Single-loss” – “Multiple-loss”	639, 986	411030	< 2.2E-16
F. tularensis*	“Core” – “Single-loss”	1010, 130	83878	1.02E-07
	“Single-loss” – “Multiple-loss”	130, 56	4805.5	2.45E-04
H. influenzae	“Core” – “Single-loss”	1026, 300	172400	7.61E-04
	“Single-loss” –	300, 54	10507	2.54E-04

	“Multiple-loss”			
L. monocytogenes	“Core” – “Single-loss”	2174, 296	413100	1.01E-15
	“Single-loss” – “Multiple-loss”	296, 61	11106	2.28E-03
**MTBC***	“Core” – “Single-loss”	2665, 527	832030	9.39E-12
	“Single-loss” – “Multiple-loss”	527, 219	79458	2.35E-16
N. meningitidis	“Core” – “Single-loss”	1270, 133	120970	< 2.2E-16
	“Single-loss” – “Multiple-loss”	133, 66	5892	4.03E-05
P. aeruginosa	“Core” – “Single-loss”	4332, 652	1882900	< 2.2E-16
	“Single-loss” – “Multiple-loss”	652, 127	46673	1.15E-02
S. pneumoniae	“Core” – “Single-loss”	1202, 188	143410	1.39E-09
	“Single-loss” – “Multiple-loss”	188, 104	13243	2.60E-07
S. suis	“Core” – “Single-loss”	1056, 357	235350	1.03E-12
	“Single-loss” – “Multiple-loss”	357, 116	26656	1.62E-06
Y. pestis*	“Core” – “Single-loss”	2396, 507	762920	< 2.2E-16
	“Single-loss” – “Multiple-loss”	507, 132	38926	1.91E-03

Table S3. Comparison of differences in the dN/dS values between the “core”, “single-loss” and “multiple-loss” pangene groups in the NCBI-annotated pangenomes using one-sided Mann-Whitney-Wilcoxon test. Only genes with 0.0001 < dN/dS <= 2 and tree length for dS >= 0.001 were analyzed (see Materials and Methods).

**Organism**	**Compared gene-loss groups**	**Number of pangenes in each group**	**U-test W**	**P-value**
B. cereus	“Core” – “Single-loss”	3092, 504	564172.5	< 2.2E-16
	“Single-loss” – “Multiple-loss”	504, 114	25739	4.13E-02
C. botulinum	“Core” – “Single-loss”	2194, 473	401535	5.58E-15
	“Single-loss” – “Multiple-loss”	473, 107	19247	5.44E-05
C. diphtheriae	“Core” – “Single-loss”	1543, 110	71284	2.49E-03
	“Single-loss” – “Multiple-loss”	110, 25	1097	5.80E-02
C. jejuni	“Core” – “Single-loss”	993, 299	122564	2.36E-06
	“Single-loss” – “Multiple-loss”	299, 34	3889	1.24E-02
C. pseudotuberculosis*	“Core” – “Single-loss”	1298, 257	150587	6.87E-03
	“Single-loss” – “Multiple-loss”	257, 94	9834	3.84E-03
E. coli-Shigella	“Core” – “Single-loss”	1434, 628	368533	2.52E-11
	“Single-loss” – “Multiple-loss”	628, 969	254441.5	1.56E-08
F. tularensis*	“Core” – “Single-loss”	754, 94	31158	2.80E-02
	“Single-loss” – “Multiple-loss”	94, 40	1766	2.91E-01
H. influenzae	“Core” – “Single-loss”	996, 298	142742.5	1.59E-01

	"Single-loss" – "Multiple-loss"	298, 51	5513	8.67E-04
L. monocytogenes	"Core" – "Single-loss"	2039, 284	213870	4.49E-13
	"Single-loss" – "Multiple-loss"	284, 58	5119.5	2.80E-06
N. meningitidis	"Core" – "Single-loss"	1197, 120	48728	3.06E-09
	"Single-loss" – "Multiple-loss"	120, 55	2459	3.45E-03
P. aeruginosa	"Core" – "Single-loss"	4071, 610	765266.5	< 2.2E-16
	"Single-loss" – "Multiple-loss"	610, 119	28895.5	2.15E-04
S. pneumoniae	"Core" – "Single-loss"	1116, 176	84258	1.21E-03
	"Single-loss" – "Multiple-loss"	176, 91	5926	2.50E-04
S. suis	"Core" – "Single-loss"	1016, 344	149761	3.61E-05
	"Single-loss" – "Multiple-loss"	344, 112	15082.5	2.79E-04

Table S4. Comparison of differences in the ENC' values between the "core", "single-loss" and "multiple-loss" pangene groups in the NCBI-annotated pangenomes using one-sided Mann-Whitney-Wilcoxon test. Only pangenes with all genes in the pangene group having length of at least 100 codons and relative standard deviation of ENC' values for individual genes <= 10% were analyzed (see Materials and Methods).

Organism	Compared gene-loss groups	Number of pangenes in each group	U-test W	P-value
B. cereus	"Core" – "Single-loss"	2851, 429	472920	1.73E-14
	"Single-loss" – "Multiple-loss"	429, 74	14403	1.02E-01
C. botulinum	"Core" – "Single-loss"	2072, 431	394500	6.93E-05
	"Single-loss" – "Multiple-loss"	431, 91	18037	1.15E-01
C. diphtheriae	"Core" – "Single-loss"	1537, 107	75518	7.88E-02
	"Single-loss" – "Multiple-loss"	107, 21	648	1.12E-03
C. jejuni	"Core" – "Single-loss"	1007, 246	116080	6.32E-02
	"Single-loss" – "Multiple-loss"	246, 31	3298	1.11E-01
C. pseudotuberculosis*	"Core" – "Single-loss"	1379, 268	167880	8.81E-03
	"Single-loss" – "Multiple-loss"	268, 81	9353	2.97E-02
E. coli-Shigella	"Core" – "Single-loss"	1420, 595	355790	1.10E-08
	"Single-loss" – "Multiple-loss"	595, 854	226690	2.39E-04
F. tularensis*	"Core" – "Single-loss"	953, 112	46461	1.25E-02
	"Single-loss" – "Multiple-loss"	112, 41	1956	8.10E-02
H. influenzae	"Core" – "Single-	957, 286	123400	5.79E-03

	loss”			
	“Single-loss” – “Multiple-loss”	286, 48	4868	6.33E-04
L. monocytogenes	“Core” – “Single-loss”	2011, 279	225860	6.38E-08
	“Single-loss” – “Multiple-loss”	279, 47	5438	3.07E-02
**MTBC***	“Core” – “Single-loss”	2657, 389	491420	5.87E-02
	“Single-loss” – “Multiple-loss”	389, 158	18878	7.49E-13
N. meningitidis	“Core” – “Single-loss”	1190, 114	49319	7.21E-07
	“Single-loss” – “Multiple-loss”	114, 43	1971	2.96E-02
P. aeruginosa	“Core” – “Single-loss”	4139, 550	795900	< 2.2E-16
	“Single-loss” – “Multiple-loss”	550, 94	21152	2.42E-03
S. pneumoniae	“Core” – “Single-loss”	1113, 168	77073	1.20E-04
	“Single-loss” – “Multiple-loss”	168, 73	4184	4.50E-05
S. suis	“Core” – “Single-loss”	968, 328	131288	1.38E-06
	“Single-loss” – “Multiple-loss”	328, 102	14333	1.45E-02
Y. pestis*	“Core” – “Single-loss”	2390, 501	515670	5.11E-07
	“Single-loss” – “Multiple-loss”	501, 124	25852	1.91E-03

Table S5. Results of correlation analysis between number of gene losses and dS along phylogenetic tree branches in the NCBI-annotated pangenomes. dS per branch was calculated as an average of dS values for individual "core" pangenes on given branch.

**Organism**	**Spearman's Rho**	**P-value**
B. cereus	0.4979	0.0394
C. botulinum	0.4615	0.1236
C. diphtheriae	0.7263	0.0066
C. jejuni	0.2914	0.2670
*C. pseudotuberculosis**	0.6370	0.0052
E. coli-Shigella	0.2769	0.0066
E. coli	0.2580	0.0188
Shigella spp.	0.8374	0.0026
*F. tularensis**	0.6347	0.0434
H. influenzae	0.1455	0.6456
L. monocytogenes	0.4574	0.0094
N. meningitidis	0.3099	0.1616
P. aeruginosa	0.0857	0.7414
S. pneumoniae	0.4284	0.0182
S. suis	0.5385	0.0160

Table S6. Differences in the amount of "core" ("unlost) and "near core" ("lost") pangenes found in each COG category. Categories in which statistically significant difference between "lost" and "unlost" pangenes was found across all species are marked by (*). (T) alongside *p-value* identifies that exact *p-value* for Mann-Whitney test couldn't be calculated due to ties (identical values) in the original data. In particular, for A, B, W, Y and Z categories statistical analysis had little to none power since in most genomes no pangenes were found in these categories.

**COG category**	**Category description**	**Number of species in which:**			**P-value**
		**"Unlost" > "Lost"**	**"Lost" > "Unlost"**	**No pangenes found in COG category**	
A*	RNA processing and modification	8	2	5	0.0272(T)
B	Chromatin structure and dynamics	3	0	12	0.0797(T)
C	Energy production and conversion	9	6	0	0.3046
D*	Cell cycle control, cell division, chromosome partitioning	15	0	0	0.0037(T)
E	Amino acid transport and metabolism	12	3	0	0.1873
F*	Nucleotide transport and metabolism	14	1	0	0.0008
G	Carbohydrate transport and metabolism	4	11	0	0.2496
H*	Coenzyme transport and metabolism	12	3	0	0.0023
I	Lipid transport and metabolism	13	2	0	0.1261
J*	Translation, ribosomal structure and biogenesis	13	2	0	0.0235
K	Transcription	3	12	0	0.1485
L*	Replication, recombination and repair	13	2	0	0.0023

M	Cell wall/membrane/envelope biogenesis	9	6	0	0.6827
N	Cell motility	4	11	0	0.6628(T)
O	Posttranslational modification, protein turnover, chaperones	10	5	0	0.1873
P	Inorganic ion transport and metabolism	5	10	0	0.3046
Q	Secondary metabolites biosynthesis, transport and catabolism	7	8	0	0.4124
R	General function prediction only	6	9	0	0.2854
S*	Function unknown	2	13	0	0.0164
T	Signal transduction mechanisms	6	9	0	0.4363
U	Intracellular trafficking, secretion, and vesicular transport	9	6	0	0.3835(T)
V*	Defense mechanisms	2	13	0	0.0023
W	Extracellular structures	1	2	12	0.6093(T)
X	Mobilome: prophages, transposons	5	7	3	0.9317(T)
Y	Nuclear structure	0	0	15	N/A
Z	Cytoskeleton	3	0	12	0.0797(T)

Table S7. Strains removed from further analysis.

Campylobacter jejuni	
**Accession number**	**Strain Name**
NC_002163	Campylobacter jejuni subsp. jejuni NCTC 11168 = ATCC 700819
NC_022351	Campylobacter jejuni subsp. jejuni 00-2538
NC_022362	Campylobacter jejuni subsp. jejuni 00-2425
NC_021834	Campylobacter jejuni 32488
Clostridium botulinum	
**Accession number**	**Strain Name**
NC_010674	Clostridium botulinum B str. Eklund 17B
NC_010723	Clostridium botulinum E3 str. Alaska E43
NC_015425	Clostridium botulinum BKT015925
Escherichia coli-Shigella **Group**	
**Accession number**	**Strain Name**
NC_017644	Escherichia coli NA114
NC_017632	Escherichia coli UM146
NC_017634	Escherichia coli O83:H1 str. NRG 857C
NC_022912	Shigella dysenteriae 1617
NC_020518	Escherichia coli str. K-12 substr. MDS42
NC_010473	Escherichia coli str. K-12 substr. DH10B
NC_017651	Escherichia coli str. 'clone D i2'
Listeria monocytogenes	
**Accession number**	**Strain Name**
NC_017728	Listeria monocytogenes 07PF0776
NC_020557	Listeria monocytogenes La111
NC_020558	Listeria monocytogenes N53-1
NC_021830	Listeria monocytogenes J1-220
Mycobacterium Tuberculosis **Cluster**	
**Accession number**	**Strain Name**
NC_017026	Mycobacterium tuberculosis RGTB327
NC_017528	Mycobacterium tuberculosis RGTB423

NC_021054	Mycobacterium tuberculosis str. Beijing/NITR203
NC_021192	Mycobacterium tuberculosis str. Haarlem/NITR202
NC_021193	Mycobacterium tuberculosis CAS/NITR204
NC_021194	Mycobacterium tuberculosis EAI5/NITR206
Pseudomonas aeruginosa	
**Accession number**	**Strain Name**
NC_022360	Pseudomonas aeruginosa c7447m
NC_022806	Pseudomonas aeruginosa PA1R
NC_022594	Pseudomonas aeruginosa PAO1-VE13
NC_022591	Pseudomonas aeruginosa PAO1-VE2
NC_022361	Pseudomonas aeruginosa PAO581

Table S8. Number of “core”, “single-loss” and “multiple-loss” pangenes in the NCBI-annotation based pangenome that were tested in each analysis. Within each investigated species all strains were compared in the pairwise manner. For each strain pair percent of orthologous genes sharing at least 80% of protein sequence similarity was calculated (see Materials and Methods).

Organism	% of highly similar orthologous gene pairs	Loss category	Total number of pangenes	Evolutionary conservation analysis	dN/dS analysis	ENC' analysis
B. cereus	94.9% - 99.96%	"Core" pangenes	3165	3165	3092	2851
		"Single-loss" pangenes	512	512	504	429
		"Multiple-loss" pangenes	118	118	114	74
		**Total "near-core" pangenes**	**630**	--	--	--
C. botulinum	96.98% - 100%	"Core" pangenes	2270	2270	2194	2072
		"Single-loss" pangenes	479	479	473	431
		"Multiple-loss" pangenes	112	112	107	91
		**Total "near-core" pangenes**	**591**	--	--	--
C. diphtheriae	98.19% - 99.95%	"Core" pangenes	1630	1630	1543	1537
		"Single-loss" pangenes	119	119	110	107
		"Multiple-loss" pangenes	26	26	25	21
		**Total "near-core" pangenes**	**145**	--	--	--
C. jejuni	96.72% - 100%	"Core" pangenes	1012	1012	993	1007
		"Single-loss" pangenes	322	322	299	246
		"Multiple-	38	38	34	31

		"Multiple-loss" pangenes				
		**Total "near-core" pangenes**	**360**	--	--	--
C. pseudotuberculosis	99.30% - 100%	"Core" pangenes	1471	1471	1298	1379
		"Single-loss" pangenes	298	298	257	268
		"Multiple-loss" pangenes	117	117	94	81
		**Total "near-core" pangenes**	**415**	--	--	--
E. coli-Shigella	96.80% - 100%	"Core" pangenes	1456	1456	1434	1420
		"Single-loss" pangenes	639	639	628	595
		"Multiple-loss" pangenes	986	986	969	854
		**Total "near-core" pangenes**	**1625**	--	--	--
F. tularensis	99.34% - 100%	"Core" pangenes	1010	1010	754	953
		"Single-loss" pangenes	130	130	94	112
		"Multiple-loss" pangenes	56	56	40	41
		**Total "near-core" pangenes**	**186**	--	--	--
H. influenzae	97.51% - 99.57%	"Core" pangenes	1026	1026	996	957
		"Single-loss" pangenes	300	300	298	286
		"Multiple-loss" pangenes	54	54	51	48
		**Total "near-core" pangenes**	**354**	--	--	--
L. monocytogenes	97.19% - 100%	"Core" pangenes	2174	2174	2039	2011
		"Single-loss" pangenes	296	296	284	279

		"Multiple-loss" pangenes	61	61	58	47
		**Total "near-core" pangenes**	**357**	--	--	--
MTBC	99.76% - 100%	"Core" pangenes	2665	2665	648	2657
		"Single-loss" pangenes	527	527	117	389
		"Multiple-loss" pangenes	219	219	37	158
		**Total "near-core" pangenes**	**746**	--	--	--
N. meningitidis	98.10% - 99.94%	"Core" pangenes	1270	1270	1197	1190
		"Single-loss" pangenes	133	133	120	114
		"Multiple-loss" pangenes	66	66	55	43
		**Total "near-core" pangenes**	**199**	--	--	--
P. aeruginosa	96.30% - 100%	"Core" pangenes	4332	4332	4071	4139
		"Single-loss" pangenes	652	652	610	550
		"Multiple-loss" pangenes	127	127	119	94
		**Total "near-core" pangenes**	**779**	--	--	--
S. pneumoniae	97.49% - 100%	"Core" pangenes	1202	1202	1116	1113
		"Single-loss" pangenes	188	188	176	168
		"Multiple-loss" pangenes	104	104	91	73
		**Total "near-core" pangenes**	**292**	--	--	--
S. suis	95.34% - 100%	"Core" pangenes	1056	1056	1016	968
		"Single-loss"	357	357	344	328

		pangenes				
		"Multiple-loss" pangenes	116	116	112	102
		**Total "near-core" pangenes**	**473**	--	--	--
Y. pestis	99.90% - 99.87%	"Core" pangenes	2396	2396	221	2390
		"Single-loss" pangenes	507	507	68	501
		"Multiple-loss" pangenes	132	132	9	124
		**Total "near-core" pangenes**	**639**	--		--
